# Feasibility of a blended therapy approach in the treatment of patients with inflammatory myopathies

**DOI:** 10.1186/s40945-021-00108-z

**Published:** 2021-05-27

**Authors:** Pierrette Baschung Pfister, Ruud H. Knols, Rob A. de Bie, Eling D. de Bruin

**Affiliations:** 1 Research and Education, Physiotherapy Occupational Therapy Research Center, University Hospital Zurich, Zurich, Switzerland; 2 Nursing and Allied Health Professions Office, Physiotherapy Occupational Therapy, University Hospital Zurich, Zurich, Switzerland; 3 Department of Epidemiology, CAPHRI Care And Public Health Research Institute, Maastricht University, Maastricht, The Netherlands; 4 Department of Health Sciences and Technology, Institute of Human Movement Sciences and Sport, ETH Zurich, Zurich, Switzerland; 5 Division of Physiotherapy, Department of Neurobiology, Care Sciences and Society, Karolinska Institutet, Stockholm, Sweden

**Keywords:** Blended therapy, Tele-rehabilitation, Exercise, Progressive resistance training, Inflammatory myopathy

## Abstract

**Background:**

Inflammatory myopathies (IMs) are a group of rare conditions characterized by proximal and often symmetrical muscle weakness and reduced muscle endurance. The recommended medical treatment is based on corticosteroids in combination with immunosuppressants. This anti-inflammatory therapy serves to inhibit and prevent inflammation but does not influence impaired muscle strength. Exercise, particularly progressive resistance training, plays therefore an important role in IMs management. Blended therapy, a combination of face-to-face treatment and telerehabilitation, may be a powerful therapy option in improving exercise program adherence in these patients.

**Methods:**

The feasibility of a 12-week interactive tablet-based home exercise program combined with face-to-face therapy sessions – a ‘blended therapy’ approach - was evaluated using a quasi-experimental one-group pre-post comparison design. Primary outcomes were recruitment, attrition and adherence rates, plus measures of acceptance (Technology Acceptance Model Questionnaire (TAM)) and satisfaction (satisfaction questionnaire). Secondary outcomes comprised potential effects of the intervention on muscle strength and function, activity limitation, disability and health-related quality of life.

**Results:**

Thirteen of the included 14 participants completed the study without any related adverse events. Mean adherence to exercise program was 84% (range: 25–100%) and participants indicated high acceptance of the intervention with mean TAM scores between 6.1 and 6.5 points. Overall satisfaction with the therapy sessions, the home program, and the technology was good. Approximately half the participants wished for longer training periods and more training sessions per week. There were inconsistent effects on muscle strength, muscle function, activity limitation, disability, and health-related quality of life.

**Conclusion:**

Blended therapy combining the use of an interactive tablet-based resistance training program with face-to-face therapy sessions is feasible and safe and participants` acceptance with this approach was high. Furthermore, results were obtained that might be useful in selecting appropriate assessments and sample sizes in future trials.

**Trial registration:**

NCT03713151.

**Supplementary Information:**

The online version contains supplementary material available at 10.1186/s40945-021-00108-z.

## Background

Inflammatory myopathies (IMs) are composed of rare and heterogeneous subgroups of neuromuscular disorders characterized by proximal and often symmetric muscle weakness and low muscle endurance, progressing over a period of weeks or months [1, 2]. The recommended medical treatment is based on high-dose corticosteroids in combination with immunosuppressive agents [3]. However, the effects of pharmacological therapy on restoring muscle strength and endurance are limited, as anti-inflammatory therapy can only inhibit and prevent inflammation but cannot directly restore damaged muscle fibres. Therefore, exercise plays a pivotal role in restoring muscle strength and performance in adult patients with IM at all disease stages [4–6]. Furthermore, there is evidence for beneficial effects of supervised and home-based exercise programs on disease activity, impairment, activity limitation and quality of life [7, 8].

Despite these encouraging effects seen in patients with IM who do exercise, there are several barriers to start and maintain an exercise program for these patients. In addition to muscle weakness and low endurance caused by chronic inflammation of skeletal muscle and microangiopathy with loss of capillaries, patients with IM often suffer from fatigue and pain [9, 10]. Besides these disease-intrinsic features, the tendency of patients with IM to avoid strenuous physical activity further exacerbates the physical deconditioning process. This vicious circle is even more aggravated by the common catabolic side effect of corticosteroids on muscle metabolism and may be reinforced by the patient’s fear of disease exacerbation [11]. This fear may be explained by the fact that until the 1990s, patients with an inflammatory disease were discouraged from exercise, assuming that it would increase inflammation [12]. However, recent studies have shown that exercise is safe and may even have an anti-inflammatory effect in patients with an inflammatory rheumatic disease [13, 14]. There are some uncertainties regarding exercise adherence in these populations. A recently published systematic review revealed that only 29 and 36% of included studies reported performed type of exercise and frequency, respectively. No information was provided concerning time and intensity of the completed training sessions [15]. Furthermore, problems with sustained adherence to home based exercise programs were also reported in clinical experience in patients with IM [16].

Blended therapy may be a powerful approach to improve adherence to an exercise program, because conventional face-to-face care and telerehabilitation (TR) are combined [17, 18]. TR, defined as the provision of remote rehabilitation services via telecommunication technologies [19], may optimize the timing, intensity and sequencing of interventions. It provides the opportunity to receive rehabilitation at home [20] as a complement to conventional face-to-face sessions. Promising results of a blended approach were achieved in supporting older adults performing an exercise program [21], and in varied rehabilitation settings such as post anterior cruciate ligament reconstruction [22] and hip and knee osteoarthritis [23].

We hypothesised that a TR approach might support patients with IM to sustain an individual exercise program while being remotely monitored and coached. To the best of our knowledge, a TR program especially designed for patients with IM does not currently exist. For this reason we developed a tablet-based exercise application (called Dividat Fit), tailored for patients with IM, which considered the best available evidence [24]. The aims of this study were [1] to evaluate the feasibility of our newly developed blended therapy approach, combining a tablet-based exercise application (app) with face-to-face physiotherapy sessions in patients with IM, and [2] to evaluate potential impacts on muscle strength, muscle function, activity limitation, disability and health-related quality of life (HRQOL). We adopted the NHR (National Institute for Health Research Evaluation) definition of a feasibility trial [25], stating “Feasibility Studies are pieces of research done before a main study in order to answer the question “Can this study be done?”, focusing on assessment of the intervention process [26].

## Methods

### Study design

All participants took part in the study for a period of 12 weeks using a quasi-experimental one-group pre-post comparison design. The study was performed from February to November 2019. The study protocol was approved by the Cantonal Ethics Committee of Zurich (Protocol No. 2018–00970) and conforms with the Declaration of Helsinki. All participants were informed about the study procedure prior to participation and signed informed consent forms. All instruction sessions and measurements were performed at the University Hospital Zurich, Switzerland.

### Participants

The study was designed for patients with the diagnosis of IM. Patients were considered eligible if they fulfilled all of the following inclusion criteria: (1) diagnosis of an IM (Polymyositis (PM) or Dermatomyositis (DM) or IM associated disorders), (2) age over 18, (3) able to walk 20 m without walking aids, (4) not currently participating in other resistance training, (5) maintained a stable medical regimen for 4 weeks prior to study initiation and (5) considered capable of maintaining a stable regimen for the course of the study. Criteria for exclusion were: (1) clinically significant concomitant disease states (e.g. renal failure, hepatic dysfunction, severe cardiovascular and/or pulmonary disease, severe osteoporosis, pulmonary hypertension, pain syndrome, paresis), (2) contraindications to physical exercise (e.g. acute exacerbation of inflammation), (3) known or suspected non-compliance, (4) drug or alcohol abuse, (5) inability to follow the procedures of the study (e.g. due to language problems, psychological disorders, dementia), (6) participation in an investigational drug study within the 30 days preceding and during the current study, (7) known pregnancy or breastfeeding, and (8) intention to become pregnant during the course of the study.

### Sample size considerations

For testing the feasibility of our new blended therapy approach, a relatively small sample was deemed appropriate [27]. For pilots and feasibility studies a rule-of-thumb advice lies around 12 participants [28]. We chose, therefore, to recruit 10–15 patients with IM.

### Recruitment

The recruitment process was performed stepwise. In a first step, a letter with the study information was sent to a random sample of 15 patients from the IM registry of the University Hospital Zurich. Interested patients could return an application form or contact the study team by phone or by e-mail. All patients who did not respond to this letter were contacted by phone and asked if they were interested in taking part in the study. Depending on the recruitment rate, in further steps another random sample of 10–15 patients was contacted. This procedure was repeated until the required number of participants was recruited. All patients who were interested and met all inclusion criteria were invited to the first assessment.

### Study intervention

**Fig. 1:**
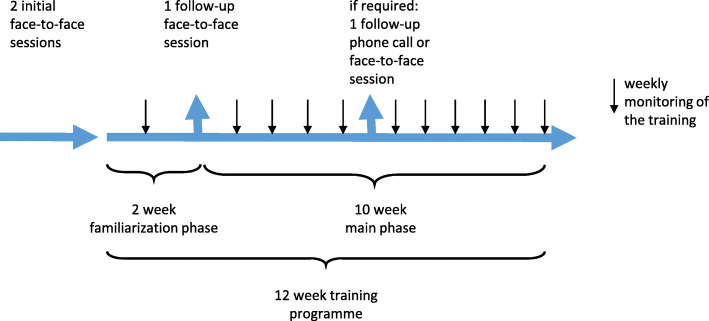
Procedure of the blended therapy

The study intervention comprised a blended therapy approach, combining face-to-face physiotherapy sessions with an interactive tablet-based exercise program. Overall, three or four 45-min individual physiotherapy sessions were planned. The exercise program consisted of a 12-week home based progressive resistance training program (Fig. 1). During the first two face-to-face sessions, the physiotherapist examined the participant, designed an individually tailored resistance training program based on that examination, demonstrated the selected exercises and then explained how to use the app. With the completion of these steps, the participant started the 12-week home-based resistance training program. After the first 2 weeks of training (familiarization phase), the third face-to-face session took place, aiming to check exercise performance and adapt the exercise program as necessary. If required, another follow-up session or phone call was added during the remaining 10 weeks of training (main phase). During the whole exercise program, the physiotherapist tele-monitored each participant weekly by checking the training diary and by adapting or commenting on the training by remote access.

Each individually tailored resistance training program was designed based on a pool of various exercises. This pool contains open and closed chain exercises for the most commonly affected muscle groups in IM [29, 30], set at different difficulty levels. An overview of the exercises is presented in Table 1.

**Table 1:** Pool of exercises of different difficulty levels

	Level 1	Level 2	Level 3	Level 4
**Bridging**	Arms beside the upper body (to stabilize)	Arms on your chest	With one leg only	
**Hip abduction**	Standing upright, abduct one leg	Lateral position, abduct one leg (knee in 90° flexion)	Lateral position, abduct one leg (knee in extension)	
**Hip extension**	Standing upright, extend one leg backwards	Support upper body on a table, extend one leg backwards	All-four position, extend one leg backwards	
**Knee extension**	Sitting on a chair, extend knee without extra weight	Sitting on a chair, extend knee with extra weight		
**Squats**	Squats in front of a table (limited ROM)	Deep squats in front of a chair (max. 90°)	Lunges	
**Heel lift**	Calf raises (with both legs)	Calf raises (with one leg)		
**Neck flexion**	Head lifts, sitting on a chair (recliner)	Head lifts, supine position	Sit-ups, supine position	
**Press-up**	Press-ups against a wall	Press-ups against a table	Press-ups (on your knees)	Press-ups (normal version)
**Shoulder abduction**	Elbows in 90°flexion (with or without extra weight)	elbows extended (with or without extra weight)	Overhead	
**Shoulder flexion**	Limited ROM, with or without extra weight	Full ROM, with or without extra weight		
**Elbow flexion**	Limited ROM, with or without extra weight	Full ROM, with or without extra weight		

Legend: *ROM* range of motion

Based on the training guidelines [24] and the individual’s personal requirements, an exercise program which included 6 to 8 exercises was tailored for each participant. Since all participants were medically stable, we aimed for the following training parameters: 2–3 sets with 8–15 repetitions at perceived intensities between levels 13 and 17 on the 6 to 20 points Borg scale [31]. Level 13 on the Borg Scale indicates “somewhat hard” intensity while levels between 15 and 17 indicate a “hard” to “very hard” intensity. This is also considered to provide an optimal strength training ‘zone’ [31]. In the familiarization phase, the intended perceived exertion was at the lower end of the recommendations (RPE Scale 13) and if the participants tolerated these intensities without pain and signs of an inflammation, the intensity was increased to 15 to 17. The progression of the exercises was adapted individually, first by increasing the amount of repetitions and sets, and then by choosing a more difficult exercise level. In addition, a personal comment could be added to each exercise.

### Training app

When participants opened the app on their tablet, they were guided through their individual exercise program in a stepwise manner. On the first screen, photos and a description of the first exercise as well as the target training parameters and personal comments from the physiotherapists were displayed. Once the first exercise was completed, participants had to record their performed repetitions, sets, perceived exertion (RPE-scale) and (if present) pain. Additionally, participants could write a brief note addressed to their physiotherapist. Dependent on the self-reported perceived exertion and pain levels, an automatically generated feedback was sent to participants: (a) if the perceived exertion is within the predefined range of intensity level, they got a positive response, (b) if the exertion is higher than the predefined range of intensity level, they were advised to exercise less, (c) if the exertion is lower than the predefined range of intensity level, they were motivated to increase the volume or intensity of the exercise. If pain is recorded, they were advised to perform the exercise correctly and if pain remains, they were prompted to contact their physiotherapist. In addition, short information about the advantages of exercise or a motivational quotation and an overview of the performed exercise parameters were provided. The participants were guided in the same way as explained above through the entire exercise program. After having finished the last exercise, an overview of the completed training session was displayed on the tablet. In this overview, participants could also see the progress they had made during the entire exercise program.

### Outcomes

The primary outcomes were the feasibility parameters of recruitment rate (and reasons for exclusion), attrition rate (and reasons for dropping out), adherence to the intervention, plus acceptance of and satisfaction with the blended therapy approach.

Acceptance was measured with a modified version of the Technology Acceptance Model (TAM) questionnaire [32]. This consists of 20 items, which are each rated on a seven-point Likert scale ranging from one (strongly disagree) to seven (completely agree), plus an open question about desired additional options for the app. The items are divided in four subgroups: perceived usefulness (seven items), perceived ease of use (six items), attitude toward using (four items), and intention to use (three items). The scores of the subgroups range from one to seven and the total score from 20 to 140.

Satisfaction was measured with a questionnaire developed by the research team, based on previous literature on program satisfaction [33]. The 30 questions were divided into four sections: individual physiotherapy sessions (five questions), home-training program (ten questions), statements about technology (three questions), and motivation (eight questions). Participants were asked to agree or disagree with several statements, using a five-point Likert scale ranging from one (agree completely) to five (disagree). In every section, participants were also invited to answer an open question.

Secondary outcomes comprised potential effects of the interventions on isometric muscle strength and muscle function, as well as on patient reported outcome measures (PROM) including activity limitation, disability, and health-related quality of life (HRQOL). All tests and questionnaires were completed before the first physiotherapy session (pre-intervention) and after the last home exercise session (post-intervention). The main goal of these outcomes was not to assess effectivity per se but see whether it was feasible from a routine collecting data point of view. The same experienced assessor, who was not involved in the intervention, performed all measurements. Isometric muscle strength was measured with Manual Muscle Testing (MMT8) and hand-held dynamometry, while muscle function was evaluated with Expanded Timed Get-up-and-Go (ETGUG), 30-s chair stand, 30-s arm curl, and functional index (FI2). Activity limitation was assessed using the Myositis Activity Profile (MAP), disability with the Stanford Health Assessment Questionnaire Disability Index (HAQ), and HRQOL with the Short Form [34] Health Survey (SF36).

The Manual Muscle Testing 8 (MMT8), an IM specific subset of the MMT, is the most commonly used strength test in IM trials [30]. MMT8 measures and scores weakness of the dominant side of eight muscle groups (shoulder abduction, elbow flexion, wrist extension, neck flexion, hip abduction and hip extension, knee extension, and ankle flexion) according to the Kendall 10-point Scale. Scores between 0 and 3, 4–6, and 7–9 indicate severe, moderate and mild weakness, respectively, and a score of 10 means that there is no detectable weakness. The single scores were added to obtain a total score varying from 0 to 80 (0 = no muscle contraction, 80 = normal strength) [29].

Isometric peak force of the same muscle groups was assessed using the MicroFET2 hand-held dynamometer (Force Evaluating and Testing, Hoggan Health Industries Inc. West Draper, UT, USA). Each muscle group was measured according to a standardised protocol [35]. The average of three measurements was used for data analysis.

The ETGUG [36] requires participants to stand up from a seated position, walk a distance of six meters at a comfortable pace, make a 180-degree turn, return to the start and sit down. The total time taken to complete the ETGUG was recorded and the gait speed was also calculated.

The 30 s chair stand measures the number of times that the participant rises to a full stand from a seated position, with arms folded, within 30 s, when moving at a comfortable pace [34].

The 30 s arm curl test measures the numbers of times a participant can lift a 1 kg weight above the head until the elbow is fully extended in a 30 s period, when moving at a comfortable pace. The number of times the weight is lifted above the head in a 30 s period is recorded for each arm, and the final score is the mean of the two measurements [34].

The functional index evaluates muscle endurance of seven muscle groups (shoulder flexion, shoulder abduction, neck flexion, hip flexion, step test, heel lift, and toe lift) [37]. For each task, participants perform as many repetitions as possible until the predefined maximal number of repetitions is reached (60 repetitions for shoulder flexion, shoulder abduction, head lift, hip flexion and step test and 120 repetitions for heel lift and toe lift). The numbers of correct repetitions achieved, following five learning repetitions, are then registered for each task (0 = severe limitation and 60 (or 120) = no limitation).

The Myositis Activity Profile (MAP) is a disease-specific activity limitation questionnaire for patients with IM [35, 38, 39]. The MAP includes 32 items which can be answered on a 7-point Likert scale; where 1 = no difficulty to perform and 7 = impossible to perform. These items are divided into four subscales (movement activities, activities of moving around, personal care and hygiene, and domestic activities) and four single items (keeping in touch with close friends and relatives, avoiding overexertion during daily activities, being able to cope with work and/or housework to a satisfactory degree, and being able to do recreational activities of choice).

The Stanford Health Assessment Questionnaire Disability Index (HAQ) consists of 20 questions divided into eight sections: dressing, rising, eating, walking, hygiene, reaching, gripping, and performing activities. Scoring within each section ranges from 0 (without any difficulty) to 3 (unable to do) [40].

The SF36 is a generic patient-reported outcome measure aimed at quantifying health-related quality of life (HRQOL). The questionnaire includes 36 questions that are divided into eight subscales: limitations in physical activities due to health problems, limitations in social activities due to physical or emotional problems, limitations in usual role activities due to physical health problems, bodily pain, general mental health (psychological distress and well-being), limitations in usual role activities due to emotional problems, vitality (energy and fatigue), and general health perceptions. The eight subscales are divided into the two main components, these being a physical component summary (PCS) and a mental component summary (MCS). Higher scores within the main components indicate a better HRQOL [41].

### Analysis

Demographic data (age, sex, BMI, diagnosis, years since diagnosis, physical activity, and medication) and the primary feasibility outcomes were reported using narrative and descriptive statistics. For attrition rate, defined as the percentage of dropouts, a 10% drop out rate was deemed acceptable. Adherence rate was calculated as the number of completed training sessions in relation to the scheduled sessions, with an adherence rate of 80% being considered acceptable [42]. Results from the satisfaction questionnaire were given as percentage of agreement with the single items. For the total TAM score, all item scores were summated. Additionally, the four subscales of the TAM were scored using the mean value of the respective item responses. Thereafter, mean, standard deviation and minimum/maximum values of the total score and the subscales were calculated.

For the secondary outcomes, descriptive statistics (mean and standard deviation for interval data and median and inter-quartile range for ordinal data) and existing floor or ceiling effects (more than 15% of the participants achieve the lowest or highest possible score) [43] were reported. The normality of the data was evaluated using the Shapiro–Wilk test. Depending on whether data were considered normally, or non-normally distributed, paired t-tests or Wilcoxon signed-rank tests were used to compare pre- and post-intervention measurements. Only data from participants who adhered to the training protocol and did not drop out were analysed (per protocol analysis). Because the focus of statistical analysis of secondary outcomes is not on significance, effect sizes (ES) were calculated for within-group differences. Since effect size depends on the dispersion, which gets artificially low when the ceiling effect is high, thus resulting in a falsely high ES, we only calculated ES for assessments without ceiling effects at baseline. For non-normal data, it is expressed as r = z/√N, where z is the approximation of the observed difference in terms of the standard normal distribution and N is the total number of samples. To enable interpretation, the following classification was assumed: 0.1 for small effect, 0.3 for medium effect, and 0.5 for large effect [44]. For normal data, Cohen’s d was calculated as follows: pre-post ES = (post-test mean – pre-test mean)/pre-test standard deviation. Here, 0.2 indicated a small, 0.5 a medium and 0.8 a large effect [45, 46]. IBM SPSS Version 25.0 software (SPSS, Inc., Chicago, IL, United States) was used for data analysis.

## Results

**Fig. 2:**
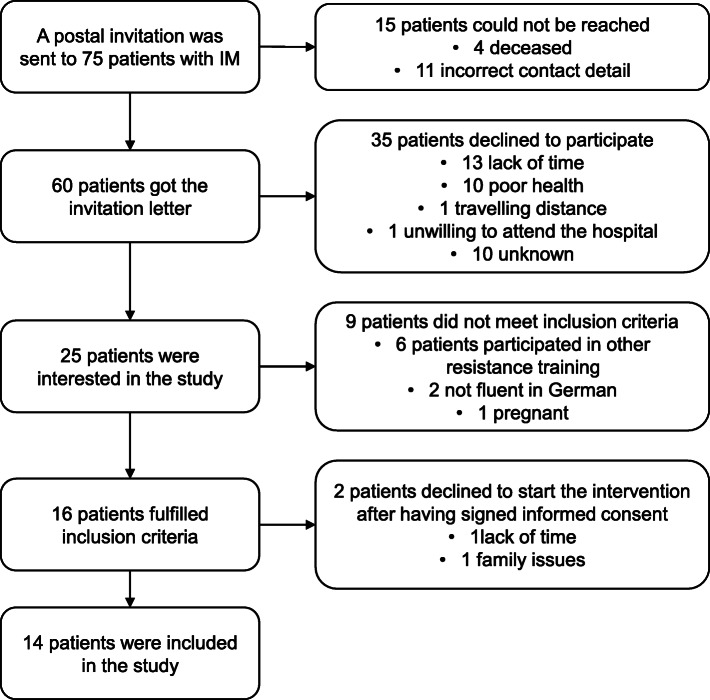
Recruitment process

Sixty out of the 75 selected patients from the IM registry could be reached and were asked whether they would be interested to participate in this study. Finally, 14 patients were included in the study, resulting in a recruitment rate of 23%. The detailed recruitment process is illustrated in Fig. 2.

Table 2 provides demographic and clinical characteristics of the participants.

**Table 2:** Demographic and clinical characteristics of the participants (N: 14)

Participant	Age	Sex	BMI	Diagnosis	Years since diagnosis	Physical activity	Medication
**1**	72	male	24	PM	4	active	steroid/immunosuppressive
**2**	40	male	28	DM	2	partially active	steroid
**3**	54	female	22	PM	28	inactive	steroid
**4**	56	female	22	DM	0.3	inactive	immunosuppressive/anti-malarial
**5**	60	male	23	DM	2	active	biologic agents
**6**	74	female	19	Statin-induced Myositis	7	active	biologic agents/immunosuppressive
**7**	55	male	17	PM	7	active	steroid/immunosuppressive
**8**	49	male	21	PM	7	active	steroid
**9**^**a**^	64	female	35	DM	13	partially active	steroid
**10**	65	female	23	Overlap Syndrome	5	partially active	–
**11**	57	female	35	Associated Myositis	7	partially active	steroid/immunosuppressive
**12**	71	female	28	Associated Myositis	6	active	steroid
**13**	54	male	27	DM	14	trained	anti-malarial
**14**	51	male	30	Anti Synthetase Syndrome	3	inactive	steroid/immunosuppressive
**Total** **Mean (SD)**	59 (10)	7 male 7 female	25 (5)	4 PM 5 DM 5 other	7.5 (6.6)	6 active 4 partially active 3 inactive 1 trained	

Legend: ^a^: dropout, *BMI* body mass index, *PM* polymyositis, *DM* dermatomyositis, *SD* standard deviation, trained: vigorous intensity activities at least three days per week, active: recommended amount of physical activity per week (150 min of moderate intense activity), partially active: certain activities but does not meet the recommended amount of physical activity per week, inactive: less than half an hour per week

One participant experienced a serious adverse event (renal colic) that was not related to the study intervention, however, was able to continue with the scheduled training. One participant dropped out because of frustration with problems manipulating the tablet, resulting in an attrition rate of 7%. Ten participants (77%) completed at least 80% of all training sessions and 8 participants completed more than the expected 24 sessions. The mean adherence rate was 84% (range 25–100%, Table 3).

**Table 3:** Overview of the performed exercises during the 12-week resistance training of every participant

Participant	Adherence rate	Bridging	Hip abd	Hip ext	Knee ext	(Adapted) Squat/lunges	Heel lift	Neck flex/sit ups	(Adapted) Press ups	Shoulder abd	Shoulder flex	Elbow flex
**1**	100	x	x	x		x	x	x	x			
**2**	100	x	x			x	x	x	x			
**3**	100	x	x		x	x			x	x		
**4**	100	x	x	x		x			x	x		
**5**	67	x	x			x	x		x			
**6**	100	x	x	x		x	x	x	x			x
**7**	88	x	x			x	x	x	x	x		x
**8**	100	x			x	x		x	x			x
**9**^**a**^	na		x		x				x		x	
**10**	100	x	x			x	x	x	x			
**11**	83		x	x		x	x	x	x		x	
**12**	29		x	x	x	x	x	x	x	x		
**13**	100	x	x	x		x	x	x				
**14**	25	x	x			x	x	x	x	x		

Legend: ^a^drop out, *abd* abduction, *ext* extension, *flex* flexion, x means that this exercise was performed, *na* not applicable

Each individual home program consisted of a warm-up session and 6–8 exercises for the hip, shoulder, knee, ankle, neck/abdomen, and elbow (Table 3). Individual training volume varied between 6 (2 × 3) repetitions and 100 (4 × 25) repetitions and perceived exertion between 10 and 16. Mean perceived exertion fell within a range of ±1 of the expected exertion in 63% of the 90 performed exercises. In 19% of the exercises, the perceived exertion was higher than planned, while in 18% it was lower. Details of performed and expected volume (repetitions and sets) as well as perceived and expected exertion of each exercise are illustrated in the Additional file 1.

### Acceptance

Table 4 presents the mean and standard deviation values for the TAM questionnaire subgroups and the scoring from each participant. In general, acceptance of this blended therapy approach was high, with mean scores of the TAM subscales between 6.1 and 6.5 and a mean total score of 128 (from a theoretical maximum of 140) points. One participant was not convinced of the usefulness of this blended approach, was reluctant to use it and stated that he would not use it again (score < 4). Participants mentioned the following desirable options for the app: “videos instead of photos”, “more space for writing messages”, “back button (to go back to the previous exercise)”, “a simple password to enter the program”, “possibility of downloading the program on the mobile phone and possibility of checking if the exercise is performed correctly (video feedback)”.

**Table 4:** Results of the Technology Acceptance Model (N: 13)

Participant	Perceived ease of use	Perceived usefulness	Attitude toward using	Intention to use	Total score
**1**	7.00	6.33	7.00	6.33	134
**2**	6.43	3.00	3.25	1.00	79
**3**	6.29	7.00	7.00	7.00	135
**4**	5.71	6.50	7.00	7.00	128
**5**	6.86	6.33	6.25	4.67	127
**6**	6.86	7.00	7.00	7.00	139
**7**	6.43	7.00	6.25	7.00	133
**8**	6.86	7.00	5.50	7.00	139
**10**	7.00	7.00	6.75	7.00	139
**11**	6.86	6.50	7.00	7.00	136
**12**	5.29	6.17	6.00	7.00	119
**13**	7.00	7.00	7.00	7.00	140
**14**	6.14	6.00	6.25	4.00	116
**Mean scores (SD)**	6.5 (0.5)	6.4 (1.1)	6.3 (1.0)	6.1 (1.8)	128 (17)

Legend: The scores of the TAM subscales may range between one to seven (mean value of the respective items) and the total score range between 20 to 140 (sum of all 20 items), *SD* standard deviation, *N* number

### Satisfaction

**Fig. 3:**
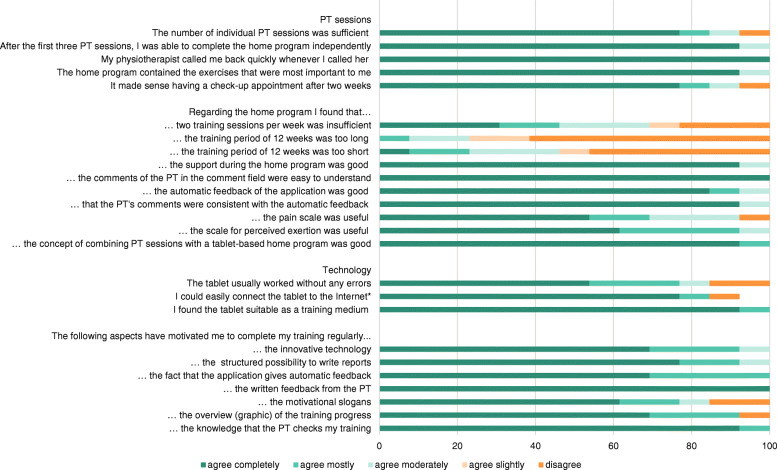
Satisfaction questionnaire

The results of the satisfaction questionnaire are illustrated in Fig. 3. More than 80% of the participants were satisfied with the number and content of the three face-to-face therapy sessions and their individual exercise programs. Two training sessions per week was deemed insufficient for most of the participants (70%) and the training period of 12 weeks was too short for 45%. Ninety percent of all participants liked the support provided during the program, the comments of the physiotherapist, the automatic feedback from the app, the scale for perceived exertion and the combination of physiotherapy sessions with the tablet-based home-program. Three participants found that the pain scale was not useful. These results were confirmed by comments from the participants. Statements from the open questions revealed that the participants were satisfied with the therapy sessions and the home program: “*I am very satisfied, feel I’m in good hands*” (participant 4), “*training at home without any problems, but maybe I would have needed one or two checks to see if I am doing the exercises correctly*” (participant 4), “*very good, because specific muscles can be trained”* (participant 9), *“was a good mix of self-check and monitoring throughout the training program”* (participant 8)*, “I think it’s a very good thing” (*participant 10*), “I find the program very good”* (participant 13*), “motivational slogans are not needed”* (participant 3*), “the control with the tablet is useful and helps ensure I really do the training; I could do the exercises at home, it does not need much space” (*participant 4*), “I think the home program is very good, I can train without equipment and save expensive gym subscriptions, and thanks to the control of the tablet I am also motivated to train*” (participant 4*).*

All participants considered the tablet a suitable training medium, although three participants mentioned that there were some technical issues with their devices. Individual statements were rather heterogeneous. Although some flaws occurred, most participants managed the tablet without any problems. Comments were: “*if you are kicked out of the program you have to enter everything all over again*” (participant 4), *“program could be improved” (*participant *7), “very good, although I am not a computer freak”* (participant 10) *and, “easy and not too complicated”* (participant 16)*.* Only one participant was overwhelmed, ceasing the training program and commenting: “*with the tablet, there always occurred situations I couldn’t manage”* (participant 12).

The most motivating aspects were perceived to be the written feedback provided by the physiotherapist, knowing that the physiotherapist monitored the training and the fact that participants were taking part in a study. Other motivational aspects were: *“I can exercise at home any time” (*participant 10)*, “The combination of the home training program and therapy sessions was perfect” (*participant 8), “*I hope that thanks to the training I will get stronger again and change the course of the disease*” (participant 4), “*supervision and training tips”* (participant 5).

### Secondary endpoints

Results of the secondary endpoints are summarized in Table 5. Since data for isometric peak force, chair stand, arm curl and ETGUG had a normal distribution parametric statistical analysis was used. All other results were presented using nonparametric statistics. Scatterplots showing the distribution of all secondary endpoints and the pattern of change are illustrated in the Additional file 2.

**Table 5:** Results of secondary endpoints before the first physiotherapy session (pre-intervention) and after the last exercise session (post-intervention), N_:_ 13

	**ceiling effect number (%)**	**pre-intervention mean (SD)**	**post-intervention mean (SD)**	**mean difference (pre-post)**	**95%CI**	***p*-value**	**effect size**
**Isometric peak force**							
Shoulder abduction (Newton)^a^	na	131 (51)	128 (49)	-2	−16 / 12	0.759	0.04
Elbow flexion (Newton)^b^	na	154 (65)	158 (54)	4	−17 / 24	0.709	0.05
Wrist extension (Newton)^a^	na	83 (32)	90 (35)	7	−3 / 16	0.169	0.20
Knee extension (Newton)^a^	na	254 (153)	258 (149)	4	−29 / 36	0.806	0.02
Ankle extension (Newton)^a^	na	141 (50)	155 (55)	15	0 / 29	0.046	0.29
Neck flexion (Newton)^a^	na	81 (37)	91 (30)	10	−3 / 23	0.107	0.28
Hip abduction (Newton)^b^	na	172 (63)	161 (46)	−11	−33 / 11	0.293	0.17
Hip extension (Newton)^b^	na	176 (62)	168 (36)	−8	−31 / 16	0.483	0.12
**Chair stand**							
Repetitions	na	11.2 (2.8)	12.6 (3.6)	1.5	0.0–2.9	0.046	0.52
**Arm curl**							
Repetitions	na	18.1 (4.2)	20.3 (7.1)	2.3	−0.7 – 5.2	0.120	0.54
**ETGUG**	na						
Overall time (seconds)	na	17.1 (2.4)	16.6 (2.4)	−0.6	−1.0 – 2.1	0.462	0.23
Speed (meter/second)	na	1.5 (0.2)	1.5 (0.3)	−0.04	−0.22 – 0.14	0.670	0.14
	**ceiling effect number (%)**	**pre-intervention median (Q1/Q3)**	**post-intervention median (Q1/Q3)**	**median difference (pre-post)**	**95%CI**	***p*-value**	**effect size**
**Manual Muscle Testing**							
Shoulder abduction (points)	11 (85)	10 (10/10)	10 (9.5/10)	0	na	0.317	na
Elbow flexion (points)	10 (77)	10 (9.5/10)	10 (10/10)	0	na	0.157	na
Wrist extension (points)	11 (85)	10 (10/10)	10 (10/10)	0	na	0.564	na
Knee extension (points)	10 (77)	10 (9/10)	10 (10/10)	0	na	0.180	na
Ankle extension (Points)	13 (100)	10 (10/10)	10 (10/10)	0	na	0.317	na
Neck flexion (points)	7 (54)	10 (7.5/10)	10 (10/10)	0	na	0.057	na
Hip abduction (points)^a^	7 (58)	10 (8/10)	10 (9.25/10)	0	na	0.214	na
Hip extension (points)^a^	6 (50)	9.5 (9/10)	10 (10/10)	0	na	0.408	na
Total score (points)^a^	4 (33)	77 (73/80)	79 (76/80)	0	na	0.514	na
**Functional Index 2**							
Shoulder abduction (repetitions)^b^	7 (64)	60 (24/60)	60 (56/60)	0	na	0.144	na
Shoulder flexion (repetitions) ^b^	7 (64)	60 (29/60)	60 (41/60)	0	na	0.715	na
Neck flexion (repetitions)^a^	2 (17)	15 (7/40)	24 (10/58)	5	na	0.021	na
Hip flexion (repetitions)^a^	2 (17)	39 (12/45)	33 (18/60)	2	na	0.333	na
Step up (repetitions)^a^	6 (50)	58 (33/60)	60 (27/60)	0	na	0.416	na
Heel lift (repetitions)^a^	5 (42)	57 (37/120)	90 (39/120)	0	na	0.173	na
Toe lift (repetitions)^a^	3 (25)	37 (20/104)	64 (25/120)	3	na	0.109	na
**SF36**							
Physical functioning (points)	1 (8)	65 (45/83)	80 (55/90)	5	na	0.219	0.24
Role-physical (points)	4 (31)	50 (0/100)	100 (0/100)	0	na	0.034	na
Bodily pain (points)	3 (23)	62 (47/87)	72 (37/100)	0	na	0.553	na
General health (points)	0	47 (39/54)	52 (44/55)	0	na	0.694	0.08
Vitality (points)	0	50 (38/70)	65 (38/78)	10	na	0.031	0.42
Social functioning (points)	4 (31)	75 (63/100)	100 (75/100)	0	na	0.107	na
Role-emotional (points)	9 (69)	100 (50/100)	100 (100/100)	0	na	0.102	na
Mental health (points)	1 (8)	76 (58/88)	80 (70/86)	0	na	0.959	0.01
Physical Component Summary	0	40 (33/47)	42 (28/51)	2	na	0.152	0.28
Mental Component Summary	0	53 (41/59)	55 (54/61)	1	na	0.152	0.28
**MAP subscales**							
Movement (points)	3 (23)	2 (1.5/3.0)	1 (1.0/3.5)	0	na	1	na
Moving around (points)	3 (23)	3 (1.5/3.0)	1 (1.0/3.0)	0	na	0.043	na
Personal care and hygiene (points)	8 (62)	1 (1.0/2.0)	1 (1.0/2.0)	0	na	0.739	na
Domestic (points) (points)	5 (38)	2 (1.0/4.0)	1 (1.0/3.5)	0	na	0160	na
**MAP single items**							
Social (points)	6 (46)	2 (1.0/3.0)	1 (1.0/2.5)	0	na	0.792	na
Avoid overexertion (points)	1 (8)	4 (2.0/4.0)	3 (1.5/4.5)	0	na	1	0
Work/school (points)	3 (23)	3 (1.5/4.0)	3 (1.0/4.0)	0	na	0.564	na
Recreational (points)	2 (15)	3 (2.0/4.0)	3 (2.5/4.0)	0	na	0.773	na
**HAQ** (points)	4 (31)	0.5 (0.0/1.0)	0.25 (0.0/0.9)	0	na	0.558	na

Legend: ^a^one missing, ^b^two missings, *Q* quartile, *%* percent, *na* not applicable, *ETGUG* Expanded Timed Get-up-and-Go, *CI* confidence interval, *%* percent, *SD* standard deviation, *HAQ* Stanford Health Assessment Questionnaire, *MAP* Myositis Activity Profile

#### Muscle strength

The MMT8 revealed ceiling effects between 50 and 100% for single muscle groups and 33% for the total score. The median difference for all single muscle groups and the total score was zero. At baseline, 12 of 14 (86%) participants demonstrated no or only mild muscle weakness (grade 7–10). Only two participants had severe muscle weakness (grade 4–6) in one muscle group each (knee extension and neck flexion). There was a slight, not significant increase in isometric peak force measured with the hand-held dynamometer in five muscle groups. Effect sizes were small (0.02–0.28). Mean strength of shoulder flexion, hip abduction and hip flexion was slightly reduced after 12 weeks of training.

#### Muscle function

On average, participants increased the amount of performed repetitions after the intervention, as measured with the chair stand and the arm curl, by 1.5 and 2.3 repetitions respectively. The difference represents a medium effect for the arm curl and the chair stand (ES: 0.54 and 0.52). The overall time to perform the ETGUG and the walking speed did not show consistent change. Data of FI2 showed ceiling effects in all muscle groups. There was a non-significant increase of the repetitions for neck flexion (median difference of five repetitions) and for toe lift (median difference of three repetitions).

#### Patient reported outcome measures

Four out of the eight subscales of the SF36 revealed ceiling effects between 23 and 69%. Only the subscales physical functioning and vitality improved slightly by 5 and 10 points, respectively. The increase of the PCS (one point) and the MCS (two points) were not significant. Floor effects between 15 and 62% were present in the subscales of the MAP (except subscale overexertion) and the HAQ. The median difference of all subscales of the MAP and the HAQ were zero.

## Discussion

This study evaluated the feasibility of a blended therapy approach, combining face-to-face sessions with a tablet-based exercise program, while also gathering data on potential impacts of the intervention on muscle strength, muscle function, activity limitation, disability and HRQL. The clinical focus was patients with IM. With an attrition rate of 7%, a mean adherence rate of 84% and no related (severe) adverse events, we demonstrated that this blended approach is feasible. Furthermore, this conclusion gains support by way of participants’ acceptance of and satisfaction with a blended therapy approach, both of which were high. The results of secondary endpoints were not conclusive: the results for muscle strength and function tests were rather mixed, and only the subscales physical functioning and vitality of the SF36 improved significantly.

### Feasibility

Out of the initial 75 potential participants, 33% were interested in joining our study. One pitfall was that our patient list was insufficiently up-to-date, so 60 patients were actually contacted. Out of those 60 patients, 25 (42%) were interested in participating in the study, resulting in 14 fulfilling the inclusion criteria and starting the exercise program. In our sample, men were over-represented. Generally, prevalence in IM is higher in females than in males [47, 48]. This known sex distribution was also seen in one of our previous studies at the University Hospital Zürich. In that study, 76% of the 50 participants were female [35].

Overall, the mean adherence rate of 84% was acceptable. This rate is comparable with the reported adherence of 79% during a 12-week resistive home exercise program with telephone support for patients with recent onset PM and DM [49], but lower than the one reported in another 12-week resistive home exercise program. In this study, one of the 12 initially included participants dropped out and 10 of the remaining 11 participants performed all of the planned 60 training sessions, resulting in an adherence rate of 95% [50]. In contrast to our study, both of these studies included recent onset patients and patients exercised 5 days a week. In our study, there were considerable differences in adherence between individuals. Two participants did not manage to exercise in a regular manner, only completing 6 and 7 of the 24 expected training sessions each during the 12-week exercise program, whereas all other participants completed at least 16 of all 24 scheduled training sessions. Our high adherence rate is in line with the high acceptance of the app indicated by the TAM questionnaire. As we had no control group with a traditional home program, we do not know whether the app influenced adherence. From other settings, we know that persuasive technologies including personalisation, self-monitoring, tailoring or goal setting, help to make exercise programs more enjoyable and therefore enhance patients’ motivation to exercise regularly [51].

Although different ideas for improvements were suggested, the participants were convinced that this app was useful and easy to use, and they felt that they would continue using it in the future. Interestingly, the two participants with low adherence rated acceptance of the app with 116 and 119 points, respectively, which was lower than the average of all other participants (128 points). Only one participant (participant 2) was even less convinced by the app. Whereas the TAM questionnaire focused mainly on the app, the satisfaction questionnaire evaluated the blended approach in its entirety. Despite the identified flaws of the app, the participants were satisfied with this tablet-based exercise program. Only one participant dropped out because she could not manipulate the app, while all other emerging technical problems could be solved without taking too much time. Participants appreciated the different motivational aspects, but interestingly, the personal contact – even though performed remotely – was judged to be the most important part. This kind of support is also emphasized in other studies. Mehra et al. described remote feedback (beside individual needs, demonstrations of functional exercises, and self-monitoring) as one of the key components of a tablet-supported exercise intervention [52] and de Vries et al. highlighted the importance of the physiotherapists’ role as facilitator by monitoring progress and rewarding adherence of the exercise program [53]. Regarding the length of the training program and the frequency of the training sessions, the participants had different opinions. Fifty percent would have preferred a longer training period and 30 % requested more training sessions. Since the guidance for prescribing exercise from the American College of Sports Medicine recommends two to three training sessions per week [54], and we wanted to avoid discouraging participants from taking part in this study by too much training, we instructed our participants to exercise at least twice a week. Exercise more than twice per week was explicitly allowed however and eight participants trained more than the minimum recommendations. With hindsight, it would have been better to recommend three training sessions a week, especially since some meta-analyses indicate that untrained individuals should do resistance training 3 days a week [55, 56]. Considering that not all patients adhere 100% to the prescribed program, the recommendation of three sessions may ensure that patients are exercising at least twice a week.

Different ideas for improvement suggested by the participants showed that our app could be developed further, but unfortunately, not all suggestions can be implemented. Since this resistance exercise program is a part of a multimodal training system, which includes exergame-driven high-intensity interval training [42] and exergame-based balance training [57], all adaptions of this app must be compatible with all parts of the system. Therefore, the development process is rather arduous, and some desirable adaptions cannot be implemented.

One important advantage of this app is that each participant must record the performed volume and intensity of each exercise. Without filling in this information, the next exercise will not be displayed in the app. In this way a detailed training diary is generated, and the physiotherapist has real-time feedback about the volume and intensity of training. So far, unreported or unknown adherence to home based exercise programs has been a weakness in many published studies [15]. Such a mandatory digital diary might be a suitable solution to overcome this issue.

We are aware of the fact that in feasibility trials an emphasis should not be placed on statistical significance [58], however, quantitative data permit the creation of sample size tables for various effect values or variance estimates [59], thus warranting the inclusion and discussion of some effect measures that may be of interest for inclusion in future randomised clinical trials.

### Muscle strength and function

The two most used assessment in patients with IM, the MMT and the FI2, both showed high ceiling effects. Therefore, they were not suitable to measure improvement in our sample. In case these measures are taken as primary outcome in a future randomised trial with patients with IM the researchers are advised to pay attention to their inclusion criteria, which should focus on strength-impaired individuals. One of the advantages of HHD, chair stand, arm curl and ETGUG is that muscle strength and function is measured with an open-ended ratio scale, without any possible ceiling effect. These assessments are, therefore, inherently capable of detecting changes over time, regardless of their extent. Nevertheless, for isometric peak force we detected only small changes. The generally small effect sizes (for improvement as well as for deterioration) indicate that maximum peak force did not change considerably. This may be explained by the fact that the participants performed dynamic progressive resistance training with a volume of between 6 and 100 repetitions and a subjective intensity of between light to somewhat hard (RPE Scale 10–16). This kind of training might have insufficient impact on maximum peak force. To improve maximum peak force, higher loads with fewer repetitions (1–6 repetitions with 80–90% of the one repetition maximum) would be more suitable [60]. However, for individuals having no experience with resistance training, such a heavy intensity is not indicated. Furthermore, maximum strength tests are highly dependent on motivation and “form of the day”, and it is uncertain whether inexperienced individuals are able to develop maximum strength [61]. Consequently, it is questionable whether a maximum strength test is suitable for this group of patients. The chair stand and arm curl seem to be more adequate to capture changes of a dynamic resistance training. Besides the advantage of the continuous, open-ended scale, they are examiner independent as well as quick and easy to perform. So far, these two promising assessments are relatively unknown and rarely used in IM trials [62]. The ETGUG was unable to detect any limitations, and our sample showed no reduction in walking speed or the overall time to perform the ETGUG as compared to healthy elderly [36]. We, therefore, conclude that this test was too easy to perform for our participants and would only recommend using the ETGUG for future IM trials.

### PROMs

The SF36 revealed modest positive effects on the physical and the mental component summary. An improvement of some aspects of HQOL was also reported in two other studies evaluating a home based resistance training program in chronic patients with IM [63, 64]. Like our own study, Alexanderson et al. identified major improvements in the subscales of physical functioning and role-physical [63]. These results indicate that resistance training mostly influences the physical parts of QOL. Another similarity between these two studies is that role-emotional has high ceiling effects and therefore appears to be a less impaired part of QOL in these samples [63]. Results of the MAP and HAQ revealed high floor effects and all participants scoring the lowest possible score at baseline cannot exhibit any further improvement.

We understand our research has some limitations. Firstly, our participants seem to have had better muscular fitness compared to many patients with IM. One reason might be that our inclusion criteria regarding physical activity were too broad. Only concomitant resistance training was prohibited and all forms of exercise other than resistance training (e.g. endurance or coordination) was allowed. Unfortunately, we recorded baseline physical activity of our participants only with categorical data. Based on these categories, only a participant who exercised three times a week is considered “trained” and we do not know how many participants exercised once or twice a week. The fact that muscular fitness was relatively high contributed to the second limitation that was the high ceiling effects for some of the secondary outcome measures, especially in the MMT8 and the FI2. These high ceiling effects affected the assessment responsiveness, since no improvements could be detected with the applied measurement methods, thus increasing the effect size by keeping the dispersion artificially low. Thirdly, our training program only contained resistance exercise, although endurance training is recommended for these patients. However, since our primary aim was to evaluate feasibility of the app, we focused on traditional resistance training only. Nevertheless, participants could perform (or continue) endurance training. It would therefore have been useful to monitor additional activities.

We included several secondary quantifiable outcome measures in our study, with which we expected to gather useful mean and variance values for some potentially important outcomes that may be used in future clinical trials. With this information, researchers might calculate necessary sample sizes for future trials. However, the approach to calculating the sample sizes required for such trials from feasibility studies, using statistical analysis, is seen with scepticism by some researchers [65]. These researchers state that clinical judgement prevails over statistical analysis for a main trial sample size calculation [65]. Finally, our study results may have been influenced by volunteer bias, assuming that our volunteer participants were more interested in technology or convinced about resistance training than the average patient. This might explain the high level of acceptance and satisfaction with both the app and the blended therapy approach.

## Conclusions

This study demonstrates that a blended therapy approach, combining a tablet-based exercise app with face-to-face physiotherapy sessions, is feasible and that this innovative approach was highly acceptable for the study participants. To further evaluate the advantages of this approach, comparison regarding adherence between traditional home programs with paper leaflets and web-based applications should be evaluated in larger randomized controlled trials. As IM is a rare disease, this would only be possible with an international multi-centre study. Based on our results, no clear conclusion about the most adequate assessment can be drawn, even though the chair stand and arm raise appear to be the most promising assessments to evaluate muscle outcomes. Measurement of maximum peak force seems to be inadequate for dynamic resistance training. To include patients’ perspectives, these assessments should be supplemented by PROMS, such as the SF36. Future exercise studies should consider whether muscle strength or muscle endurance should be exercised and adapt the dosage of sets and repetitions accordingly. Furthermore, measurement properties, especially responsiveness should be defined for all assessments.

## Supplementary Information

**Additional file 1.** Performed and expected volume (repetitions and sets) and perceived and expected exertion of each exercise.

**Additional file 2.** Scatterplots showing the distribution of all secondary endpoints and the pattern of change.

## Data Availability

The datasets used and/or analysed during the current study are available from the corresponding author on reasonable request.

## Abbreviations

app: Application
DM: Dermatomyositis
ETGUG: Expanded Timed Get-up-and-Go
FI2: Functional Index
HAQ: Stanford Health Assessment Questionnaire Disability Index
HHD: Hand-Held Dynamometry
HRQOL: Health-Related Quality of Life
IMs: Inflammatory Myopathies
MAP: Myositis Activity Profile
MMT: Manual Muscle Testing (MMT8)
PM: Polymyositis
PROM: Patient-Reported Outcome Measures
RPE: Rated Perceived Exertion
SF36: Short Form (36) Health Survey
TAM: Technology Acceptance Model
TR: Telerehabilitation

## References

[1] Dalakas MC (1991). Polymyositis, dermatomyositis and inclusion-body myositis. N Engl J Med.

[2] Bohan A, Peter JB (1975). Polymyositis and dermatomyositis (first of two parts). N Engl J Med.

[3] Gordon PA, Winer JB, Hoogendijk JE, Choy EH (2012). Immunosuppressant and immunomodulatory treatment for dermatomyositis and polymyositis. Cochrane Database Syst Rev.

[4] Lundberg IE, Vencovsky J, Alexanderson H (2014). Therapy of myositis: biological and physical. Curr Opin Rheumatol.

[5] Habers GE, Takken T (2011). Safety and efficacy of exercise training in patients with an idiopathic inflammatory myopathy--a systematic review. Rheumatology..

[6] Alexanderson H (2018). Exercise in myositis. Curr Treatm Opt Rheumatol.

[7] Alexanderson H (2016). Physical exercise as a treatment for adult and juvenile myositis. J Intern Med.

[8] Sveaas SH, Smedslund G, Hagen KB, Dagfinrud H (2017). Effect of cardiorespiratory and strength exercises on disease activity in patients with inflammatory rheumatic diseases: a systematic review and meta-analysis. Br J Sports Med.

[9] Regardt M, Basharat P, Christopher-Stine L, Sarver C, Bjorn A, Lundberg IE (2015). Patients' experience of myositis and further validation of a myositis-specific patient reported outcome measure - establishing Core domains and expanding patient input on clinical assessment in myositis. Report from OMERACT 12. J Rheumatol.

[10] Opinc AH, Brzezinska OE, Makowska JS (2019). Disability in idiopathic inflammatory myopathies: questionnaire-based study. Rheumatol Int.

[11] Loell I, Lundberg IE (2011). Can muscle regeneration fail in chronic inflammation: a weakness in inflammatory myopathies?. J Intern Med.

[12] Alexanderson H (2009). Exercise effects in patients with adult idiopathic inflammatory myopathies. Curr Opin Rheumatol.

[13] Lundberg IE, Nader GA (2008). Molecular effects of exercise in patients with inflammatory rheumatic disease. Nat Clin Pract Rheumatol.

[14] Nader GA, Lundberg IE (2009). Exercise as an anti-inflammatory intervention to combat inflammatory diseases of muscle. Curr Opin Rheumatol.

[15] Baschung Pfister P, de Bruin E, Tobler-Ammann B, Maurer B, Knols R (2015). The relevance of applying exercise training principles when designing therapeutic interventions for patients with inflammatory myopathies: a systematic review. Rheumatol Int.

[16] Alexanderson H, Lundberg IE (2012). Exercise as a therapeutic modality in patients with idiopathic inflammatory myopathies. Curr Opin Rheumatol.

[17] Verwey R, van der Weegen S, Spreeuwenberg M, Tange H, van der Weijden T, de Witte L (2014). A monitoring and feedback tool embedded in a counselling protocol to increase physical activity of patients with COPD or type 2 diabetes in primary care: study protocol of a three-arm cluster randomised controlled trial. BMC Fam Pract.

[18] Kloek CJ, Bossen D, Veenhof C, van Dongen JM, Dekker J, de Bakker DH (2014). Effectiveness and cost-effectiveness of a blended exercise intervention for patients with hip and/or knee osteoarthritis: study protocol of a randomized controlled trial. BMC Musculoskelet Disord.

[19] Russell TG (2007). Physical rehabilitation using telemedicine. J Telemed Telecare.

[20] Seelman KD, Hartman LM (2009). Telerehabilitation: policy issues and research tools. Int J Telerehabil.

[21] Mehra S, Visser B, Cila N, van den Helder J, Engelbert RH, Weijs PJ (2019). Supporting older adults in exercising with a tablet: a usability study. JMIR Hum Factors.

[22] Dunphy E, Hamilton FL, Spasic I, Button K (2017). Acceptability of a digital health intervention alongside physiotherapy to support patients following anterior cruciate ligament reconstruction. BMC Musculoskelet Disord.

[23] Kloek CJJ, Bossen D, Spreeuwenberg PM, Dekker J, de Bakker DH, Veenhof C (2018). Effectiveness of a blended physical therapist intervention in people with hip osteoarthritis, knee osteoarthritis, or both: a cluster-randomized controlled trial. Phys Ther.

[24] Baschung Pfister P, Sterkele I, Keller Trevisan C, de Bruin ED (2020). Entwicklung eines Trainingsleitfadens für Patienten mit entzündlicher Muskelerkrankung. Physioscience.

[25] Research NIfH. Feasibility 2020. Available from: https://www.nihr.ac.uk/about-us/glossary.htm?letter=F&postcategory=-1.

[26] Tickle-Degnen L (2013). Nuts and bolts of conducting feasibility studies. Am J Occup Ther.

[27] Moore CG, Carter RE, Nietert PJ, Stewart PW (2011). Recommendations for planning pilot studies in clinical and translational research. Clin Transl Sci.

[28] Julious SA (2005). Sample size of 12 per group rule of thumb for a pilot study. Pharm Stat.

[29] Harris-Love MO, Shrader JA, Koziol D, Pahlajani N, Jain M, Smith M, Cintas HL, McGarvey CL, James-Newton L, Pokrovnichka A, Moini B, Cabalar I, Lovell DJ, Wesley R, Plotz PH, Miller FW, Hicks JE, Rider LG (2009). Distribution and severity of weakness among patients with polymyositis, dermatomyositis and juvenile dermatomyositis. Rheumatology..

[30] Rider LG, Koziol D, Giannini EH, Jain MS, Smith MR, Whitney-Mahoney K, Feldman BM, Wright SJ, Lindsley CB, Pachman LM, Villalba ML, Lovell DJ, Bowyer SL, Plotz PH, Miller FW, Hicks JE (2010). Validation of manual muscle testing and a subset of eight muscles for adult and juvenile idiopathic inflammatory myopathies. Arthritis Care Res (Hoboken).

[31] Borg GA (1982). Psychophysical bases of perceived exertion. Med Sci Sports Exerc.

[32] Masrom M (2007). Technology acceptance model and e-learning. Technology..

[33] Hill J, Bird HA, Hopkins R, Lawton C, Wright V (1992). Survey of satisfaction with care in a rheumatology outpatient clinic. Ann Rheum Dis.

[34] Agarwal S, Kiely PD (2006). Two simple, reliable and valid tests of proximal muscle function, and their application to the management of idiopathic inflammatory myositis. Rheumatology..

[35] Baschung Pfister P, de Bruin ED, Sterkele I, Maurer B, de Bie RA, Knols RH (2018). Manual muscle testing and hand-held dynamometry in people with inflammatory myopathy: an intra- and interrater reliability and validity study. PLoS One.

[36] Wall JC, Bell C, Campbell S, Davis J (2000). The timed get-up-and-go test revisited: measurement of the component tasks. J Rehabil Res Dev.

[37] Alexanderson H, Broman L, Tollback A, Josefson A, Lundberg IE, Stenstrom CH (2006). Functional index-2: validity and reliability of a disease-specific measure of impairment in patients with polymyositis and dermatomyositis. Arthritis Rheum.

[38] Alexanderson H, Reed AM, Ytterberg SR (2012). The myositis activities profile -- initial validation for assessment of polymyositis/dermatomyositis in the USA. J Rheumatol.

[39] Alexanderson H, Lundberg IE, Stenstrom CH (2002). Development of the myositis activities profile--validity and reliability of a self-administered questionnaire to assess activity limitations in patients with polymyositis/dermatomyositis. J Rheumatol.

[40] Fries JF, Spitz P, Kraines RG, Holman HR (1980). Measurement of patient outcome in arthritis. Arthritis Rheum.

[41] Bullinger M (1995). German translation and psychometric testing of the SF-36 Health Survey: preliminary results from the IQOLA Project. International Quality of Life Assessment. Soc Sci Med (1982).

[42] Rebsamen S, Knols RH, Pfister PB, de Bruin ED (2019). Exergame-driven high-intensity interval training in untrained community dwelling older adults: a formative one group quasi- experimental feasibility trial. Front Physiol.

[43] Vet HCWd (2011). Measurement in medicine : a practical guide.

[44] Fritz CO, Morris PE, Richler JJ (2012). Effect size estimates: current use, calculations, and interpretation. J Exp Psychol Gen.

[45] Rhea MR (2004). Determining the magnitude of treatment effects in strength training research through the use of the effect size. J Strength Cond Res..

[46] Cohen J (1988). Statistical power analysis for the behavioral sciences.

[47] Meyer A, Meyer N, Schaeffer M, Gottenberg JE, Geny B, Sibilia J (2015). Incidence and prevalence of inflammatory myopathies: a systematic review. Rheumatology..

[48] Parker MJS, Oldroyd A, Roberts ME, Ollier WE, New RP, Cooper RG (2018). Increasing incidence of adult idiopathic inflammatory myopathies in the City of Salford, UK: a 10-year epidemiological study. Rheumatol Adv Pract.

[49] Alexanderson H, Munters LA, Dastmalchi M, Loell I, Heimburger M, Opava CH (2014). Resistive home exercise in patients with recent-onset polymyositis and dermatomyositis -- a randomized controlled single-blinded study with a 2-year followup. J Rheumatol.

[50] Alexanderson H, Stenstrom CH, Jenner G, Lundberg I (2000). The safety of a resistive home exercise program in patients with recent onset active polymyositis or dermatomyositis. Scand J Rheumatol.

[51] Fogg BJ (2008). Persuasive Technology: Using Computers to Change What We Think and Do. Morgan Kaufmann Series in Interactive Technologies B, USA (2008).

[52] Mehra S, Visser B, Dadema T, van den Helder J, Engelbert RH, Weijs PJ (2018). Translating behavior change principles into a blended exercise intervention for older adults: design study. JMIR Res Protoc.

[53] de Vries HJ, Kloek CJJ, de Bakker DH, Dekker J, Bossen D, Veenhof C (2017). Determinants of adherence to the online component of a blended intervention for patients with hip and/or knee osteoarthritis: a mixed methods study embedded in the e-exercise trial. Telemed J E Health.

[54] Garber CE, Blissmer B, Deschenes MR, Franklin BA, Lamonte MJ, Lee IM, Nieman DC, Swain DP, American College of Sports Medicine (2011). American College of Sports Medicine position stand. Quantity and quality of exercise for developing and maintaining cardiorespiratory, musculoskeletal, and neuromotor fitness in apparently healthy adults: guidance for prescribing exercise. Med Sci Sports Exerc.

[55] Peterson MD, Rhea MR, Alvar BA (2005). Applications of the dose-response for muscular strength development: a review of meta-analytic efficacy and reliability for designing training prescription. J Strength Cond Res.

[56] Rhea MR, Alvar BA, Burkett LN, Ball SD (2003). A meta-analysis to determine the dose response for strength development. Med Sci Sports Exerc.

[57] Wuest S, Borghese NA, Pirovano M, Mainetti R, van de Langenberg R, de Bruin ED (2014). Usability and effects of an Exergame-based balance training program. Games Health J.

[58] Thabane L, Ma J, Chu R, Cheng J, Ismaila A, Rios LP, Robson R, Thabane M, Giangregorio L, Goldsmith CH (2010). A tutorial on pilot studies: the what, why and how. BMC Med Res Methodol.

[59] Rogan S, Radlinger L, Schmidtbleicher D, de Bie RA, de Bruin ED (2015). Preliminary inconclusive results of a randomised double blinded cross-over pilot trial in long-term-care dwelling elderly assessing the feasibility of stochastic resonance whole-body vibration. Eur Rev Aging Phys Act.

[60] Kraemer WJ, Ratamess NA (2004). Fundamentals of resistance training: progression and exercise prescription. Med Sci Sports Exerc.

[61] Oesch P (2007). Assessments in der muskuloskelettalen Rehabilitation: Bern : Hans Huber.

[62] van der Stap DK, Rider LG, Alexanderson H, Huber AM, Gualano B, Gordon P (2016). Proposal for a candidate Core set of fitness and strength tests for patients with childhood or adult idiopathic inflammatory myopathies. J Rheumatol.

[63] Alexanderson H, Stenstrom CH, Lundberg I (1999). Safety of a home exercise programme in patients with polymyositis and dermatomyositis: a pilot study. Rheumatology..

[64] Alemo Munters L, Dastmalchi M, Andgren V, Emilson C, Bergegard J, Regardt M (2013). Improvement in health and possible reduction in disease activity using endurance exercise in patients with established polymyositis and dermatomyositis: a multicenter randomized controlled trial with a 1-year open extension followup. Arthritis Care Res (Hoboken)..

[65] Sim J (2019). Should treatment effects be estimated in pilot and feasibility studies?. Pilot Feasibility Stud.

