# Supplementary material for: Feasibility of a blended therapy approach in the treatment of patients with inflammatory myopathies
Source: Arch Physiother. 2021 May 27;11:14. doi: 10.1186/s40945-021-00108-z (PMC8157458; doi:10.1186/s40945-021-00108-z)
Supplement: Supplementary file 2 — Additional file 2. Scatterplots showing the distribution of all secondary endpoints and the pattern of change. [file 40945_2021_108_MOESM2_ESM.docx]

**Scatterplots showing the distribution of all secondary endpoints and the pattern of change**

**Results of isometric peak force measured with the hand-held dynamometer**


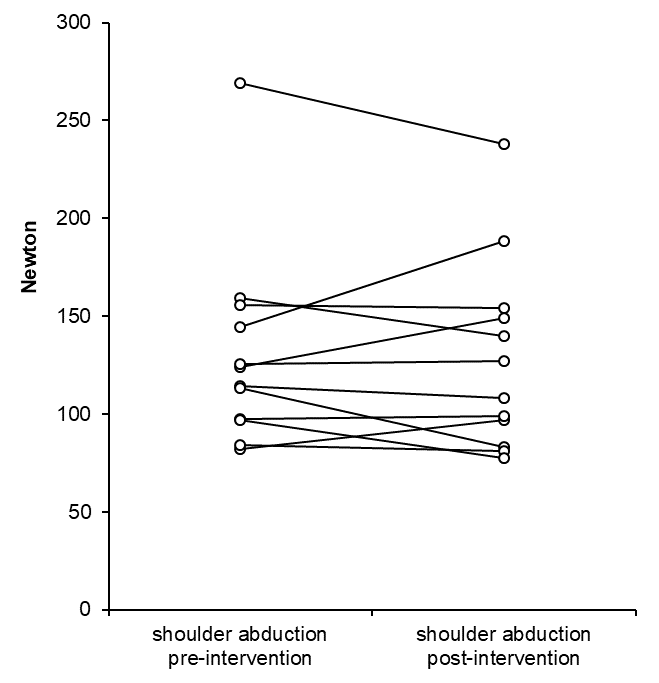

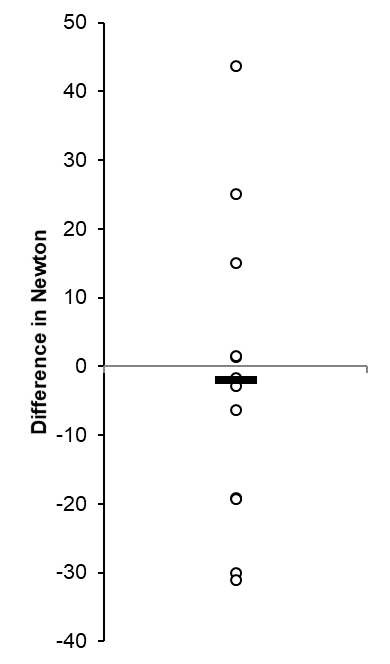

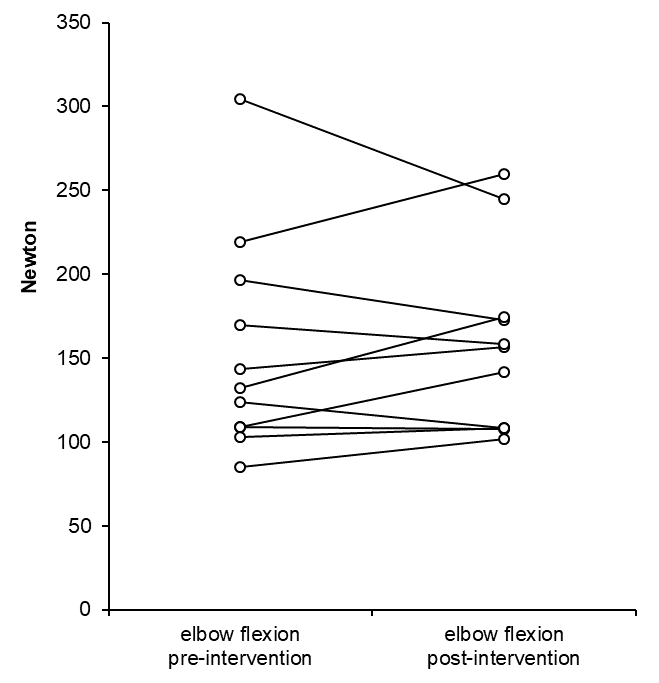

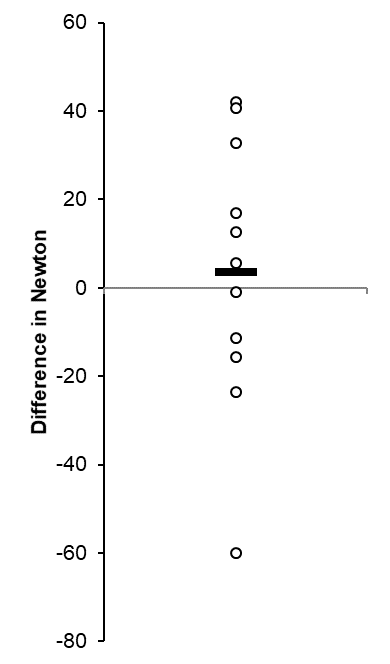


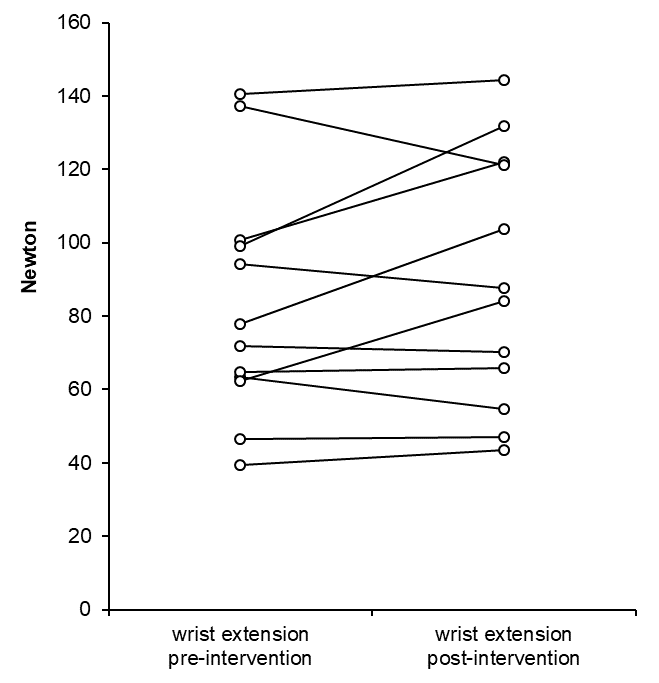

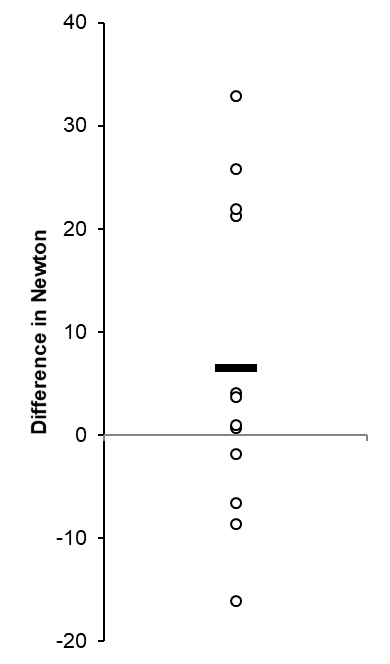

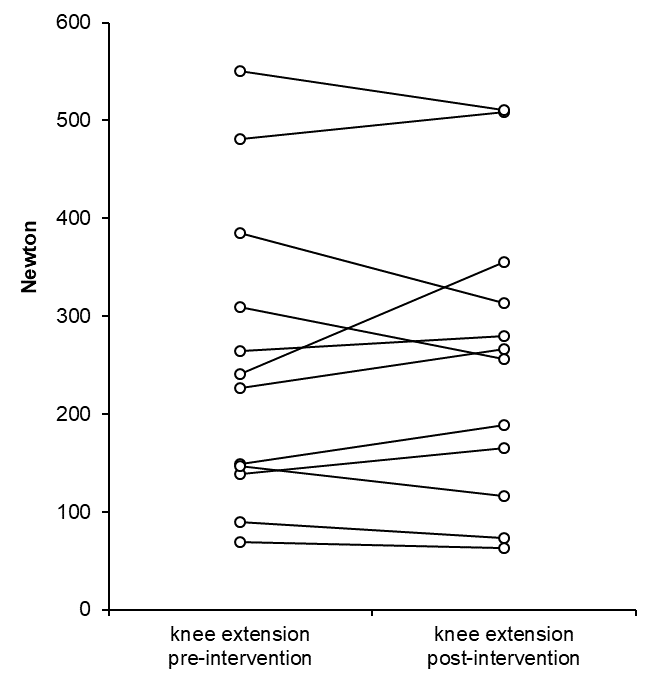

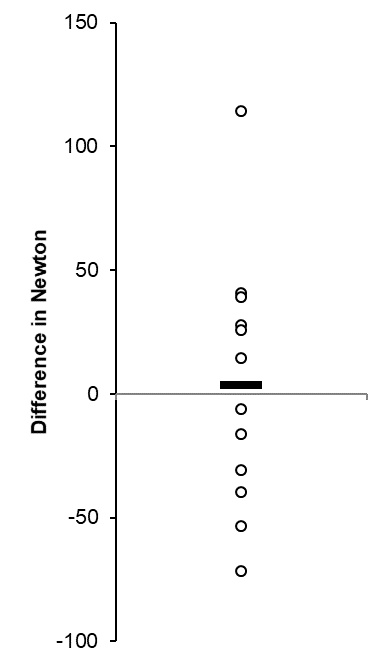


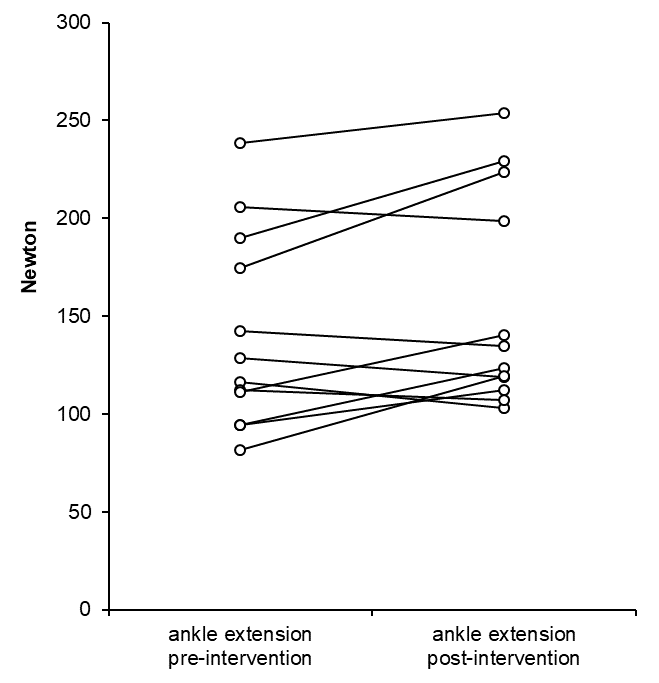

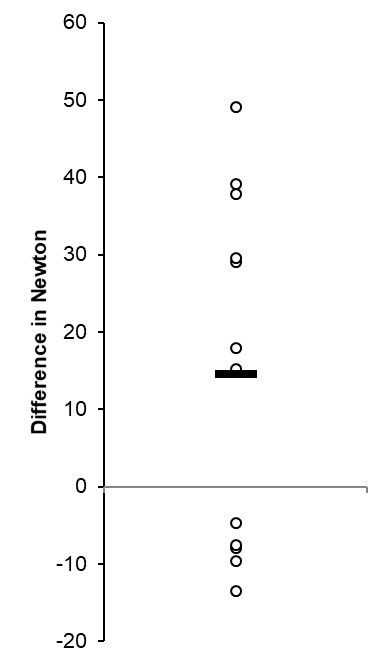

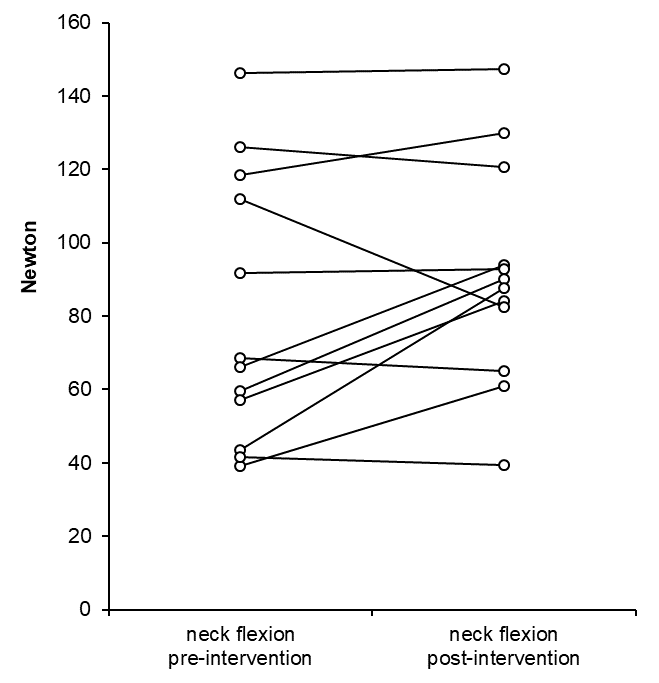

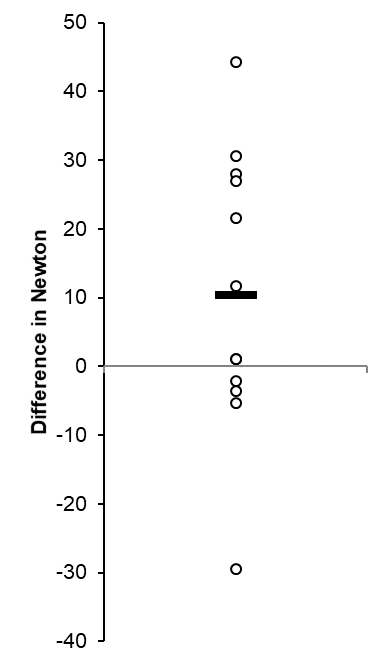


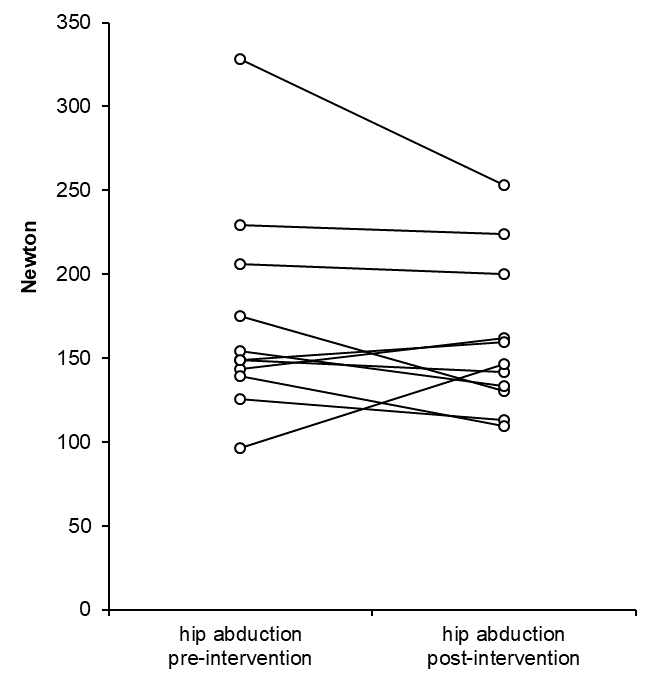

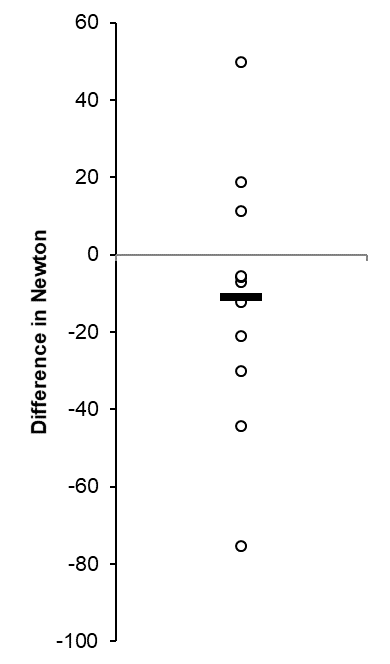

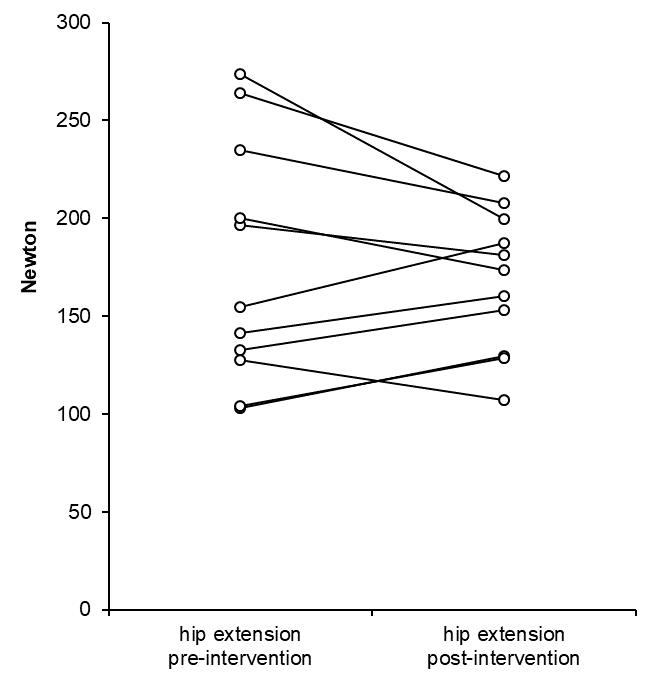

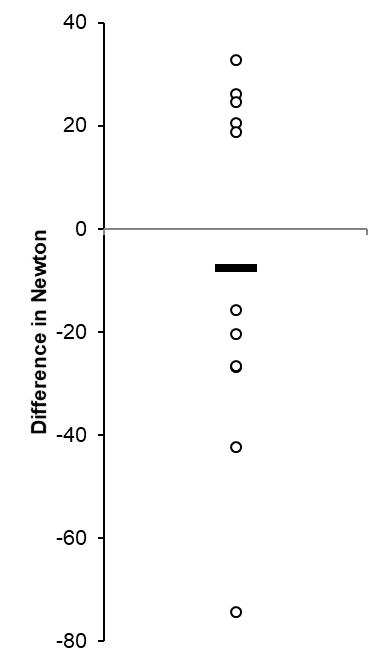


Legend: the solid lines show the mean difference.

**Results of 30-second chair stand (chair stand) and 30-second arm curl (arm curl)**


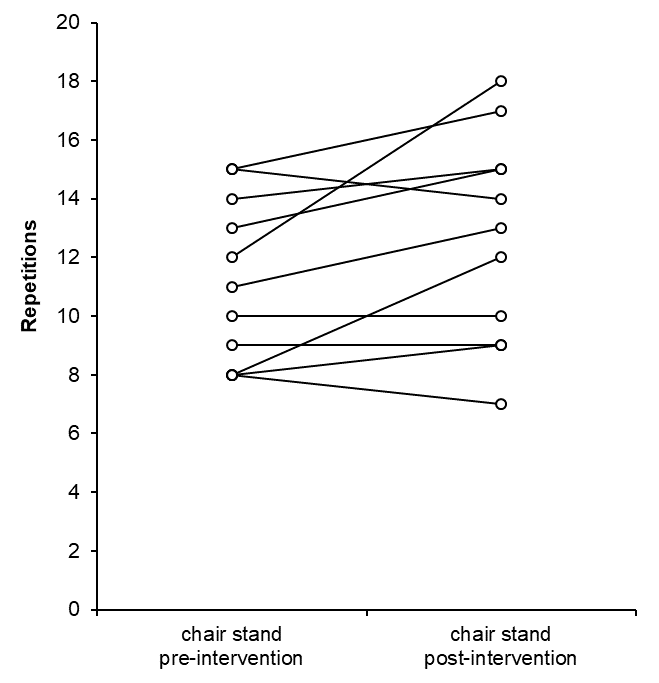

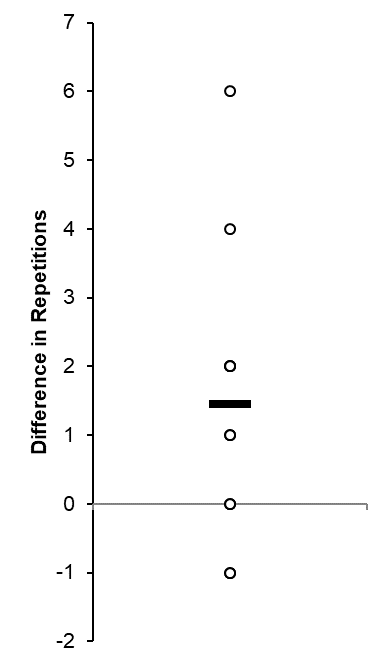

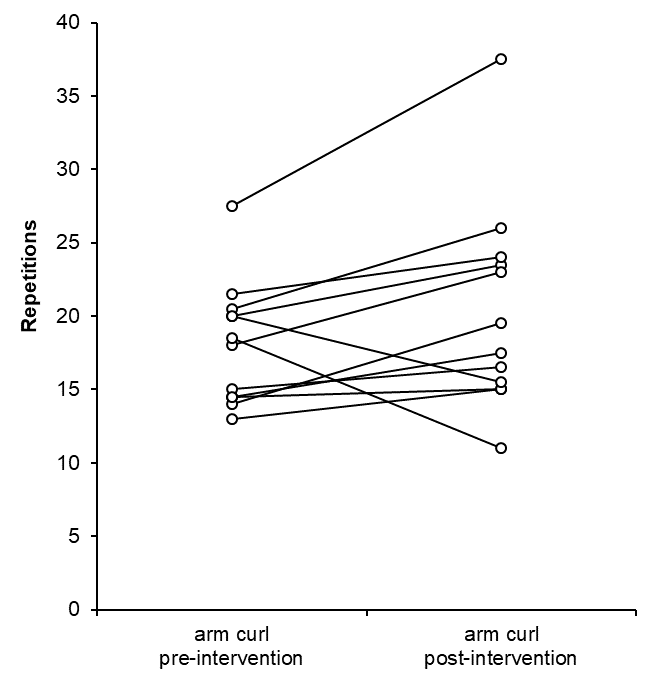

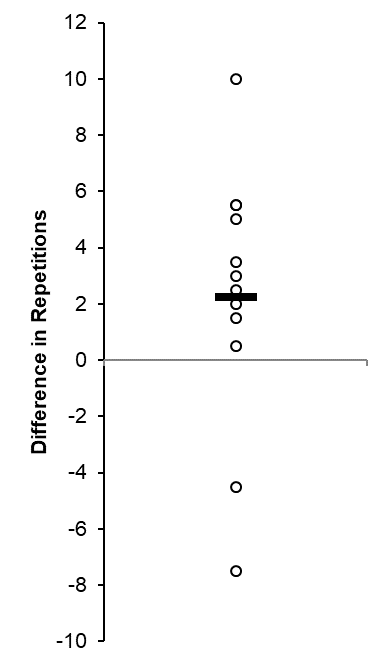


Legend: the solid lines show the mean difference.

**Results of Expanded Timed Get-up-and-Go (ETGUG)**


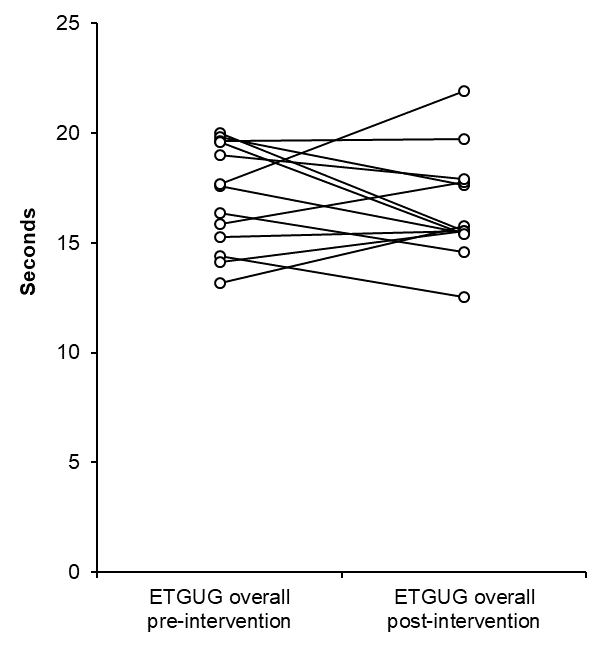

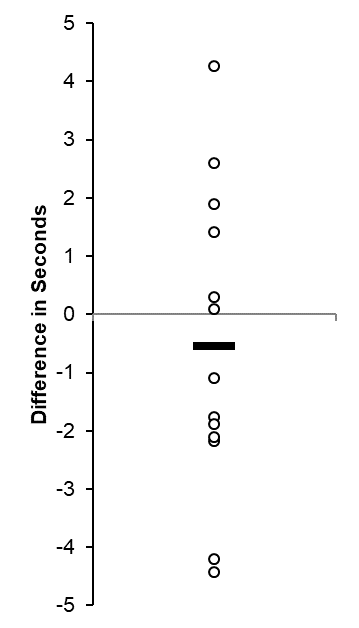

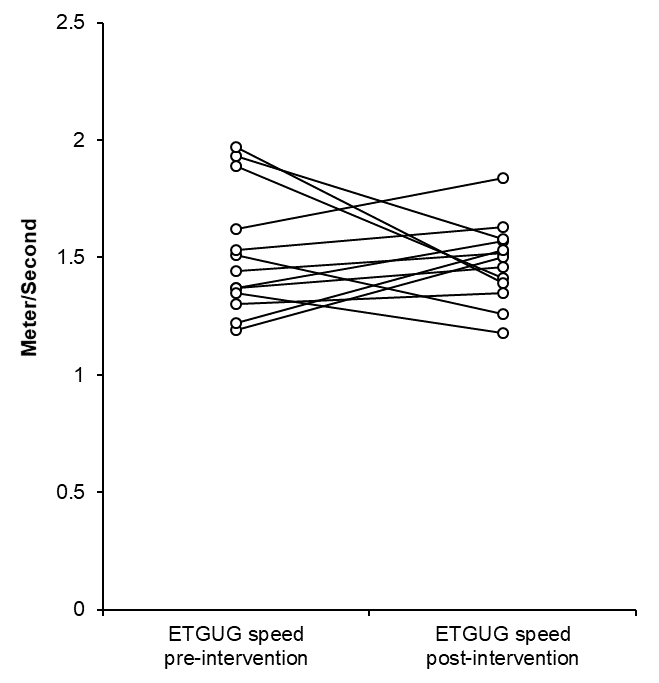

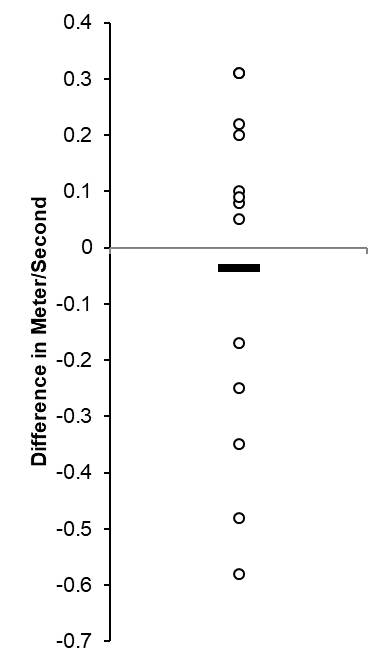


Legend: the solid lines show the mean difference.

**Results Manual Muscel Testing**


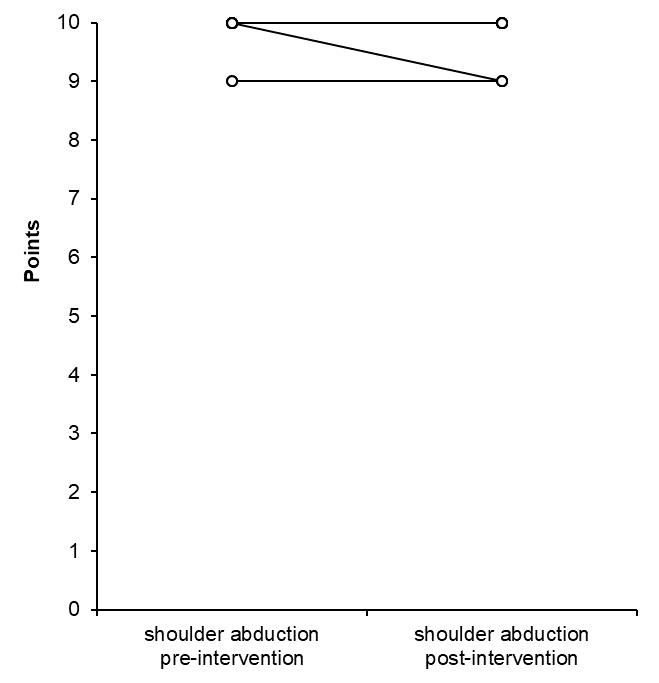

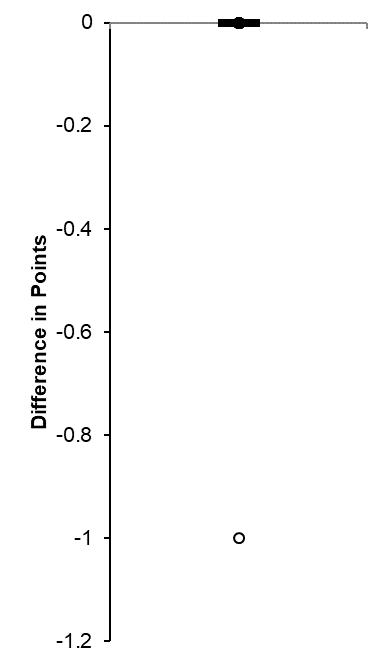

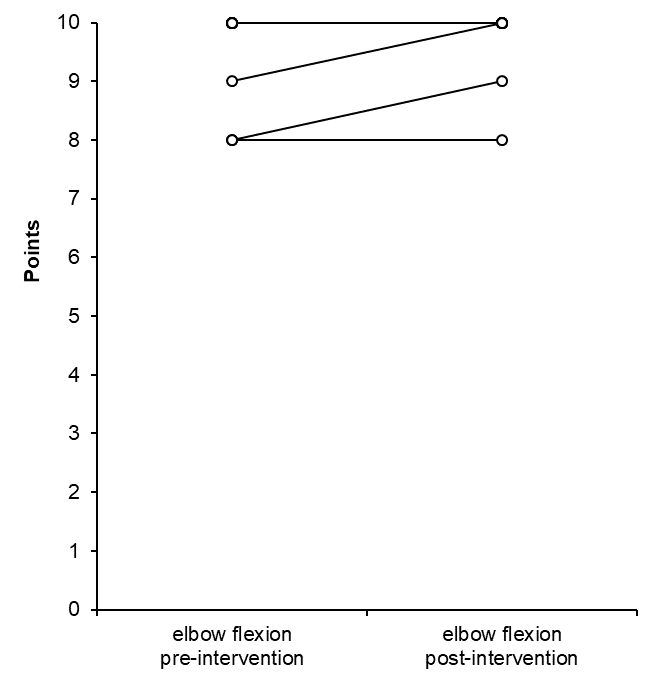

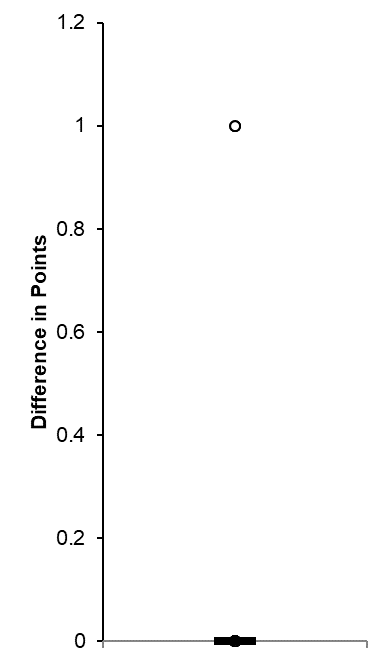


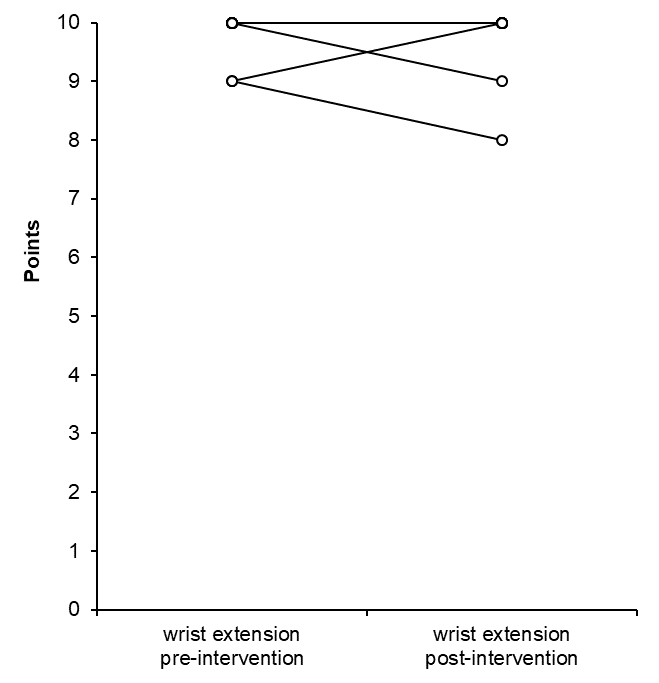

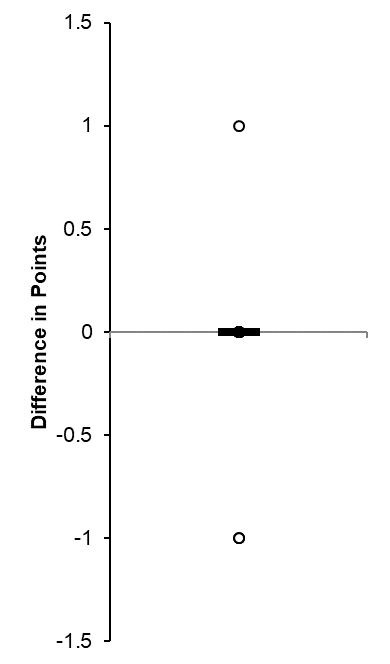

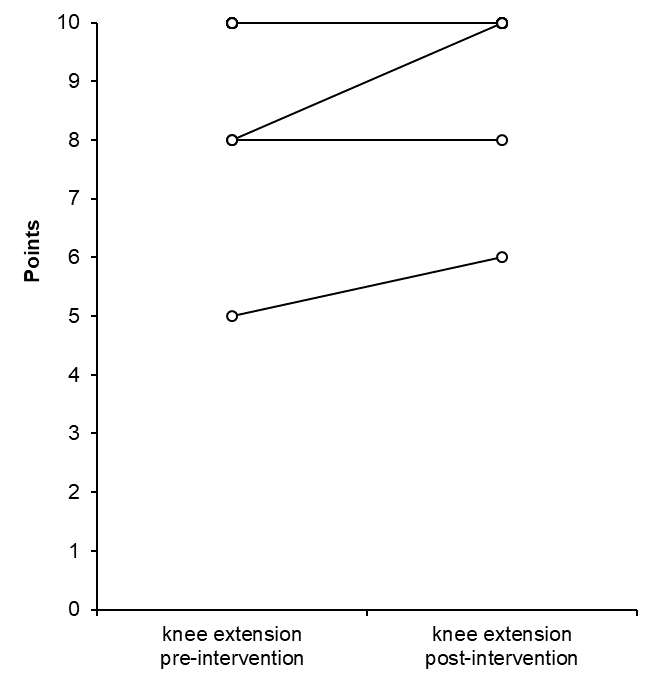

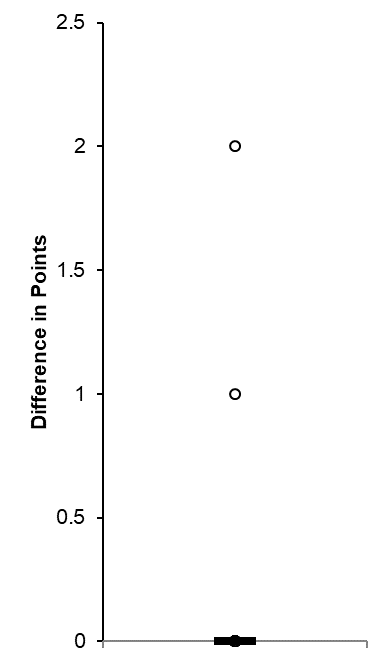


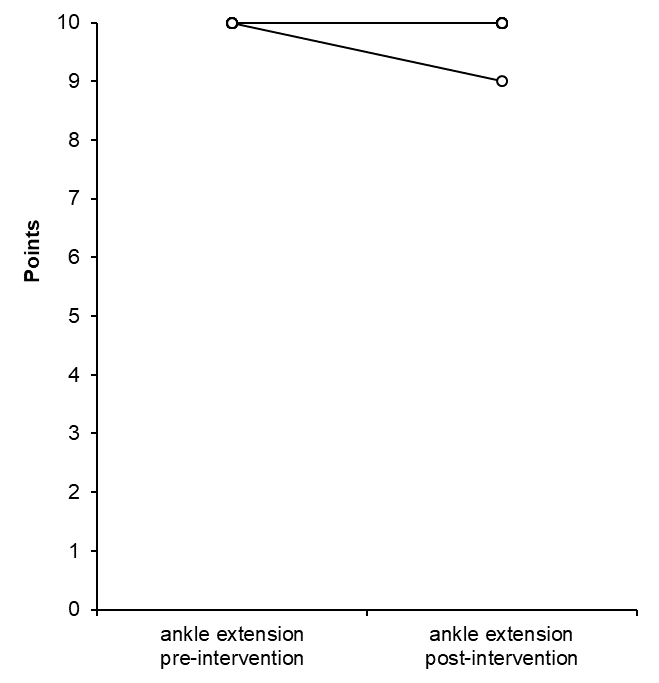

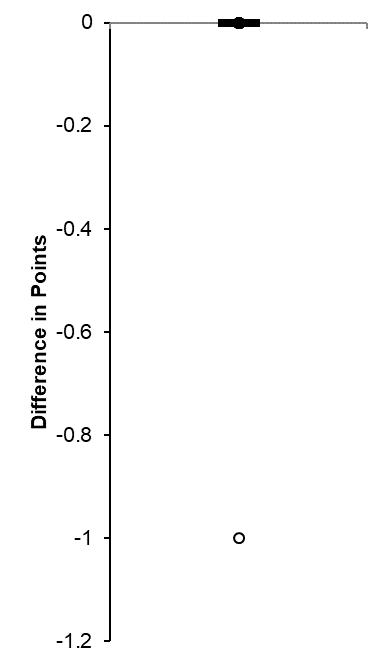

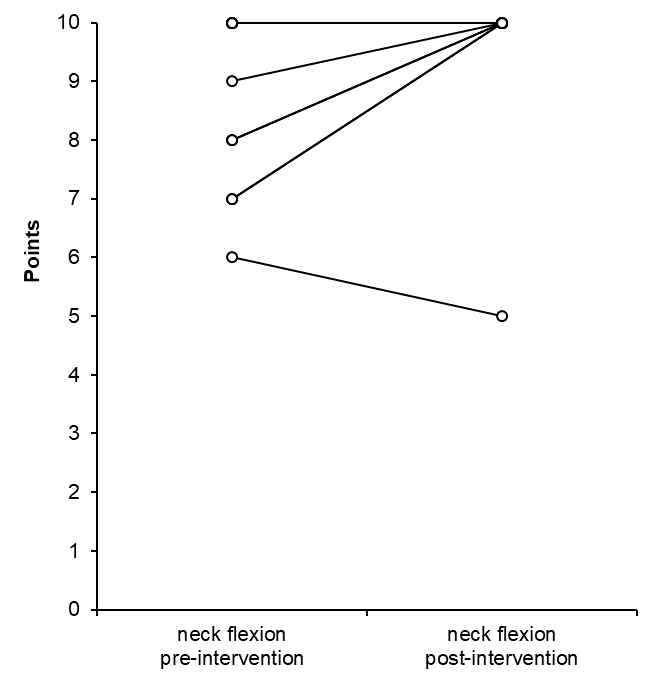

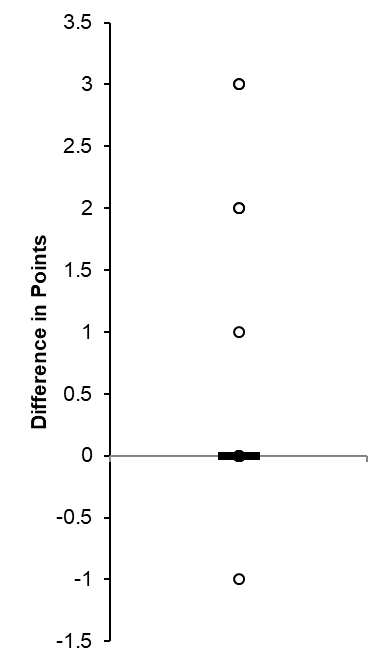


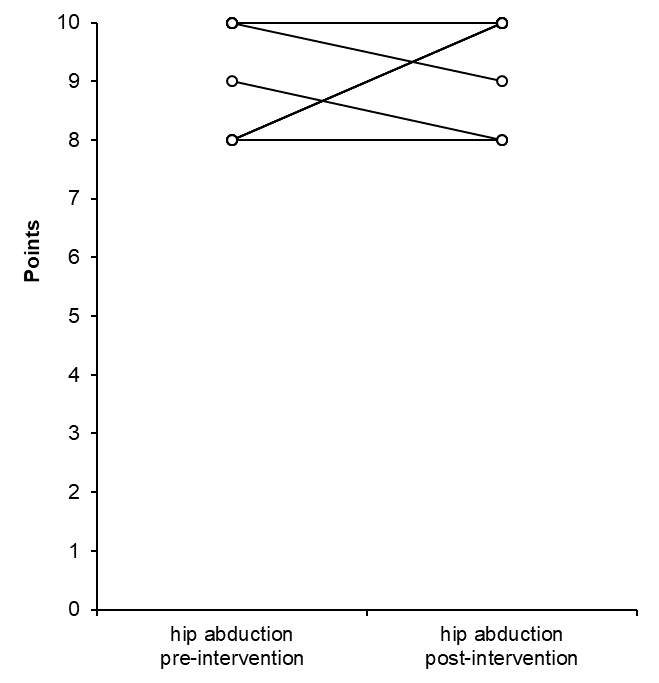

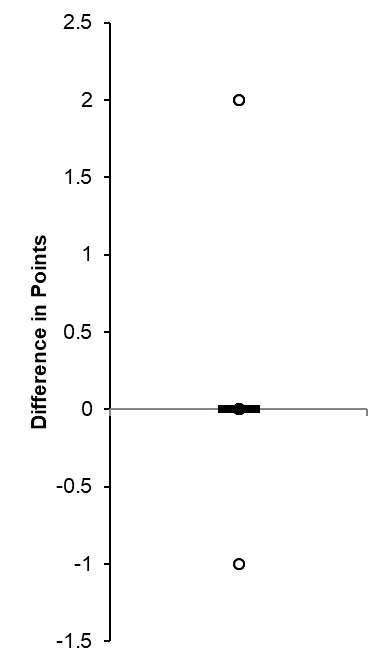

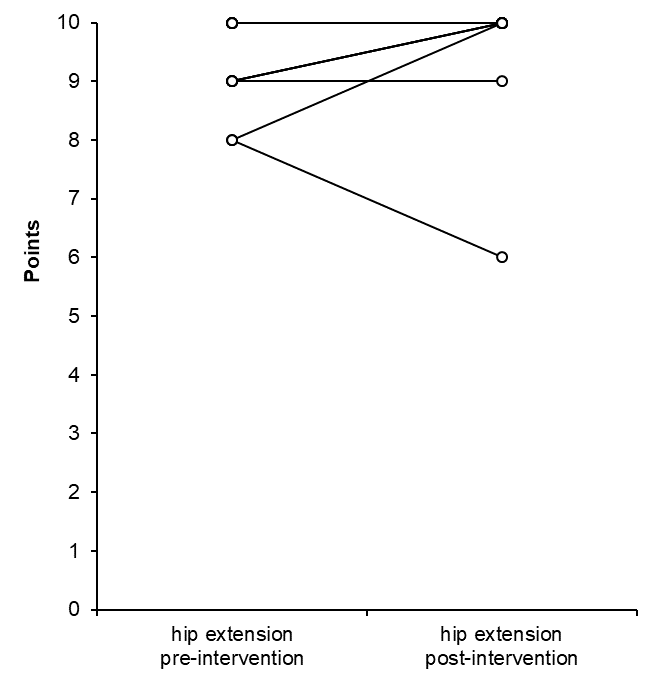

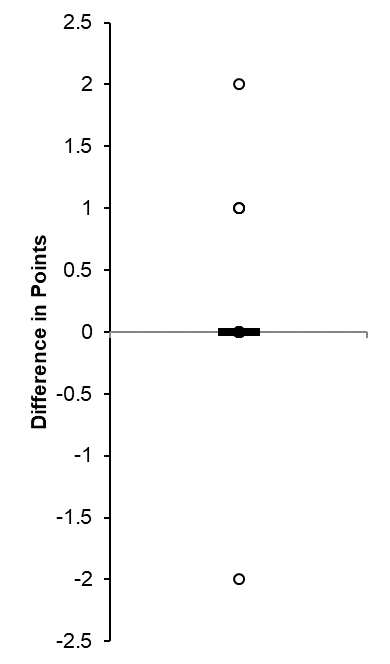


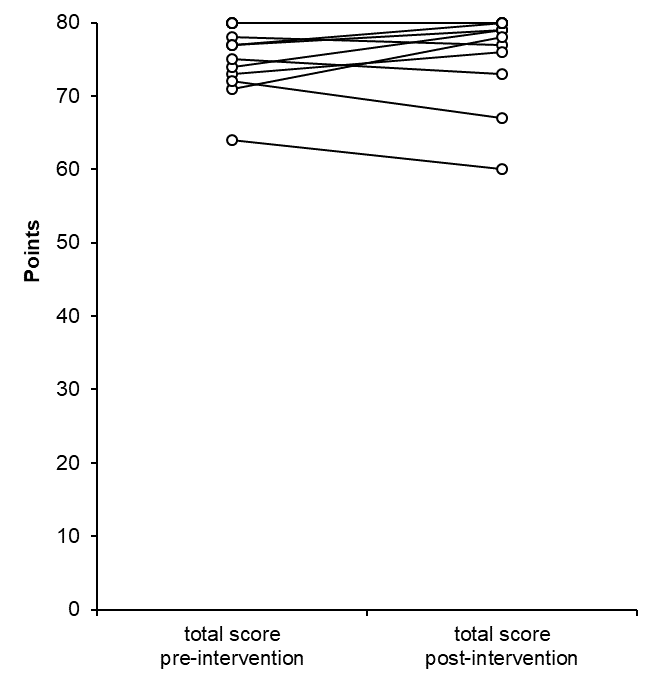

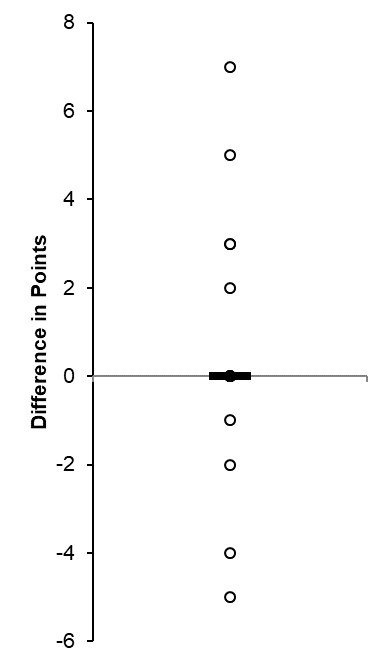


Legend: the solid lines show the median difference.

**Results o Functional Index 2**


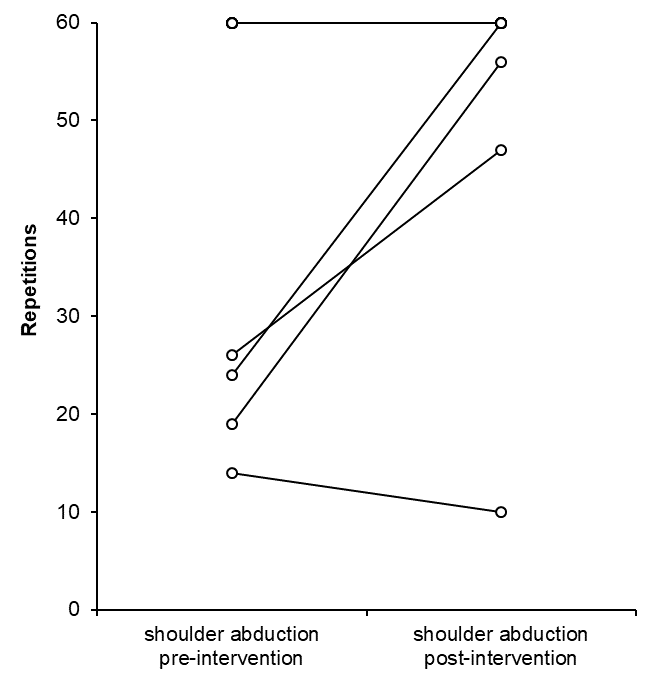

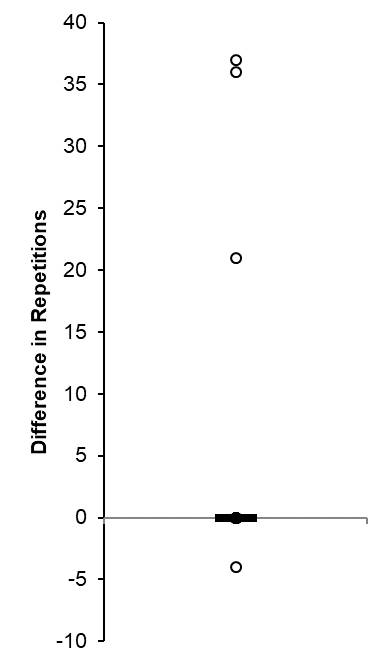

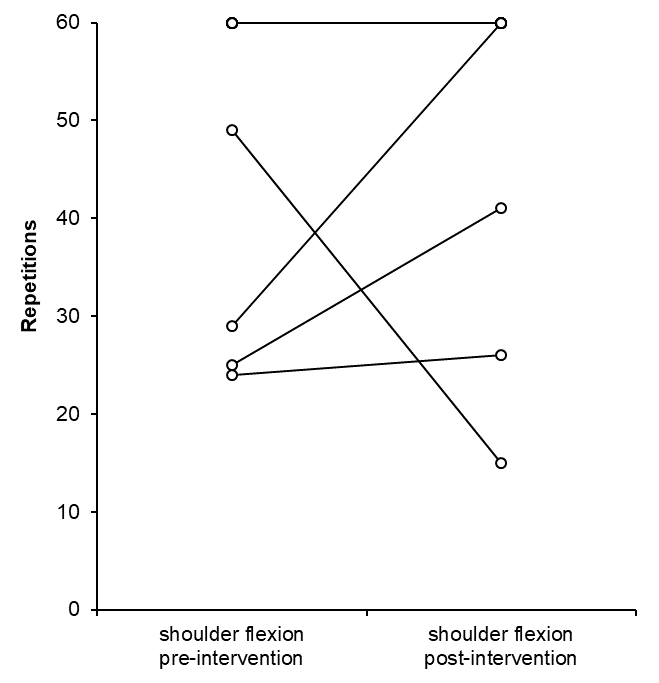

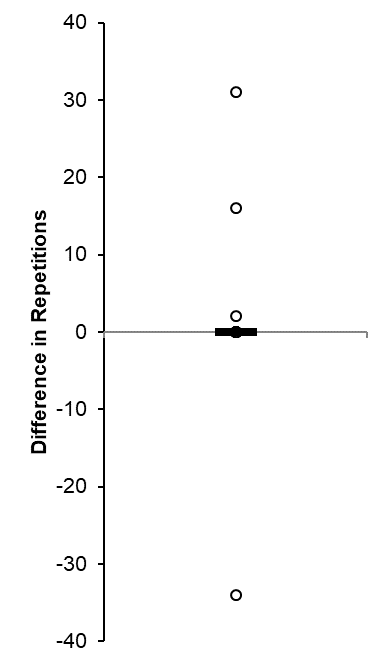


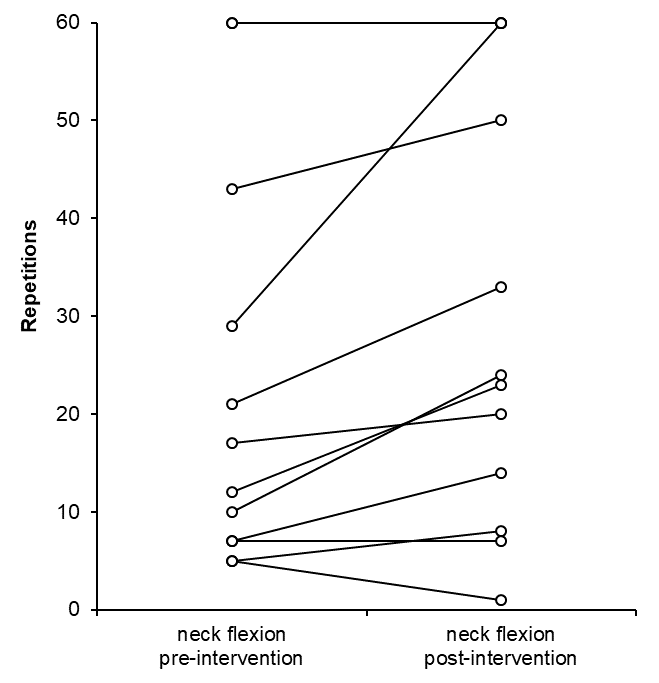

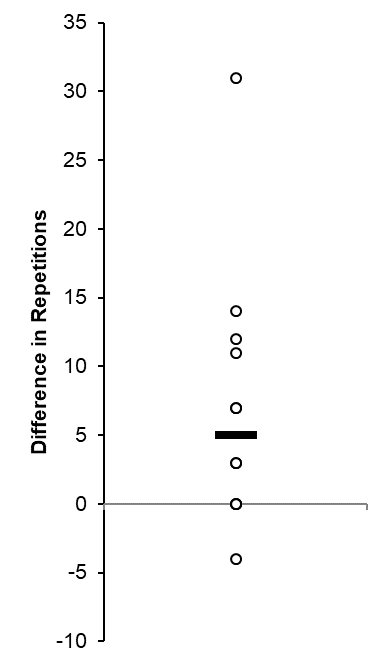

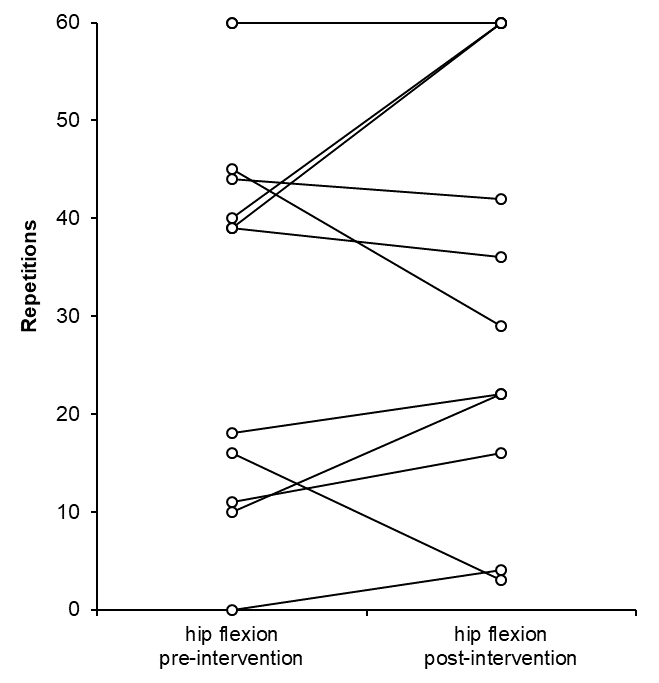

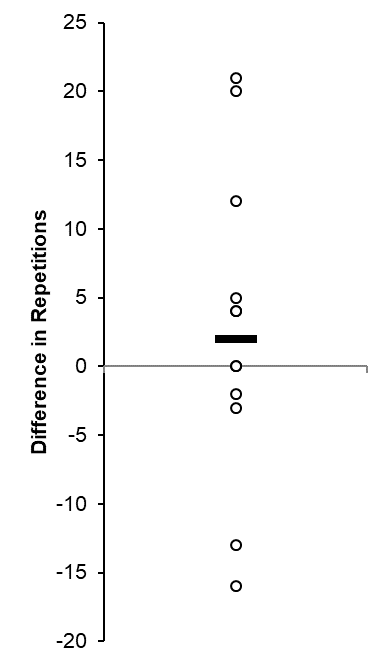


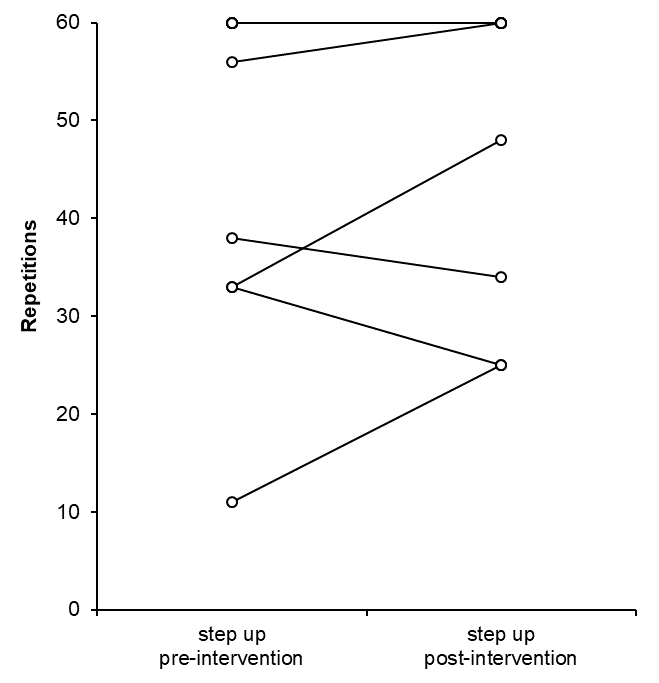

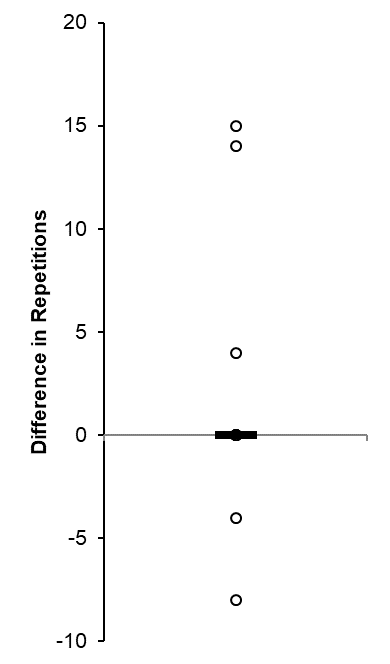


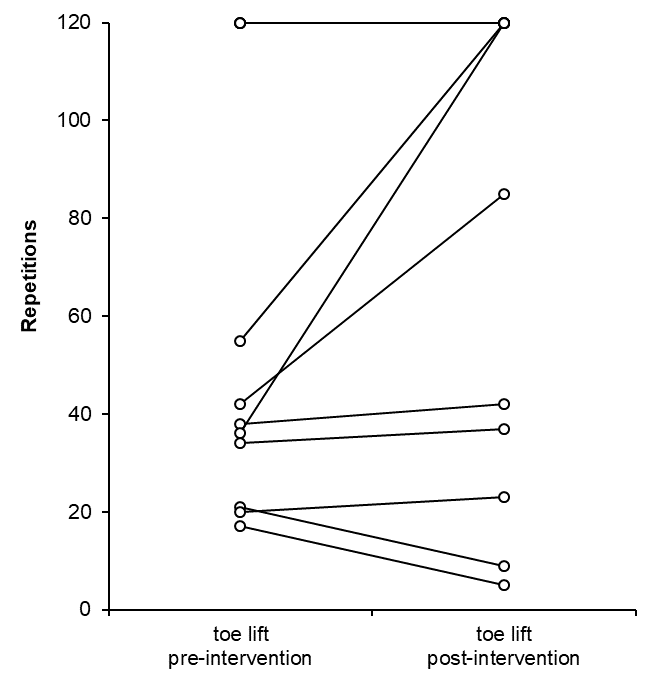

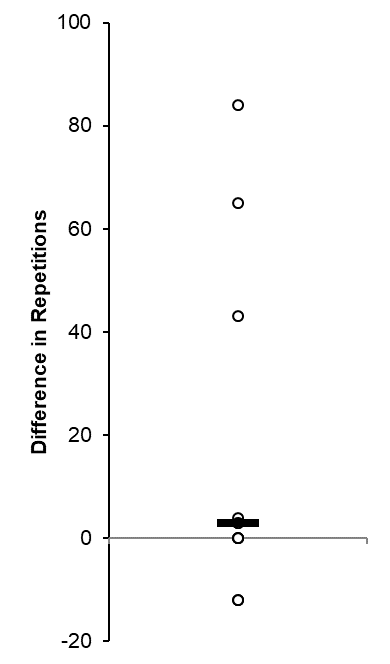

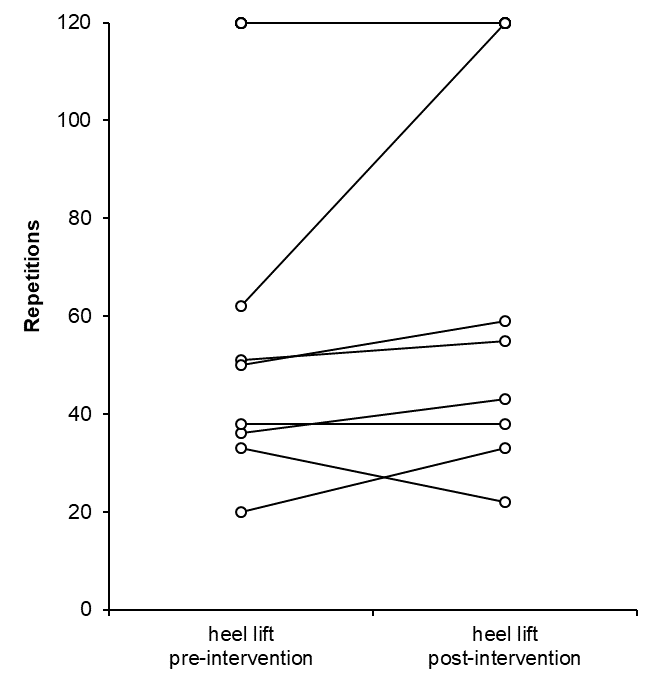

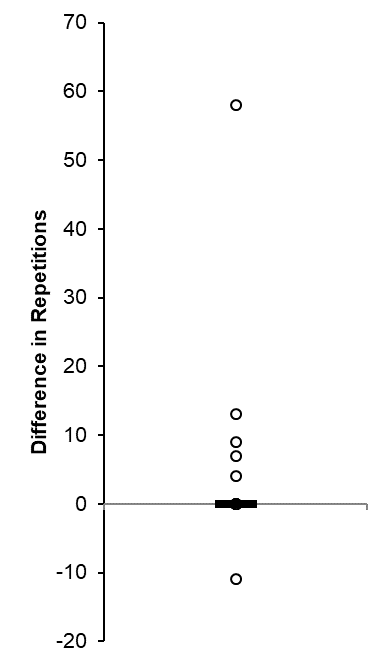


Legend: the solid lines show the median difference.

**Results of the Short Form Health Survey (SF36)**


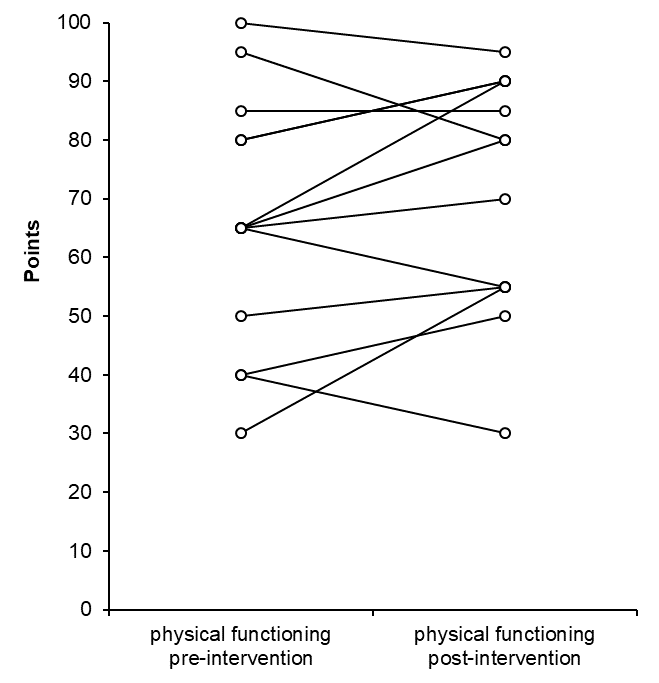

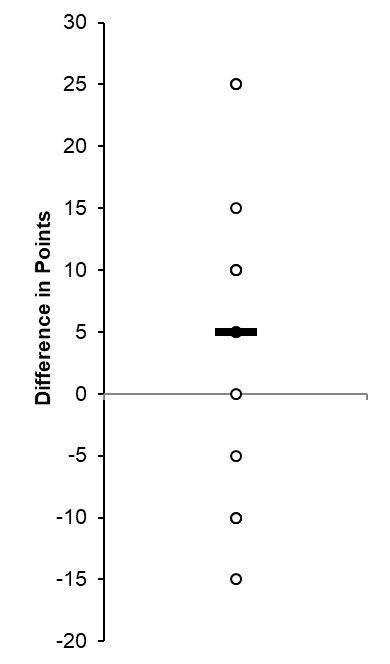

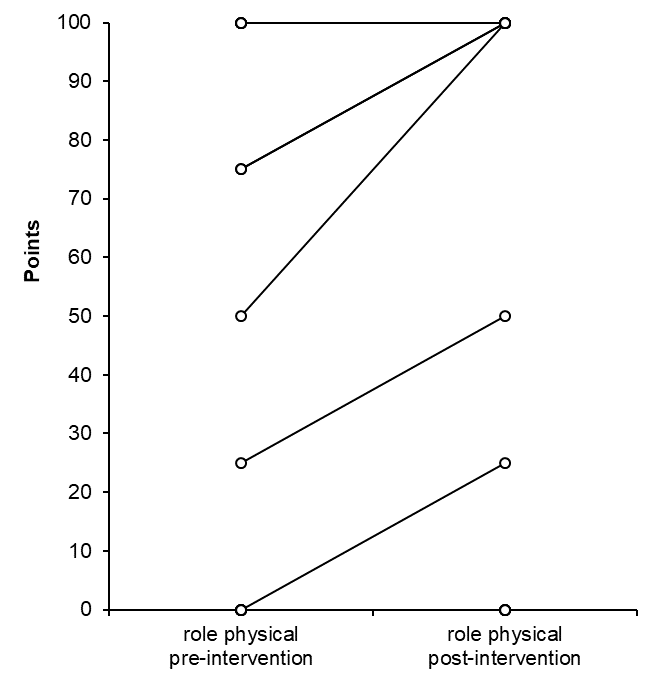

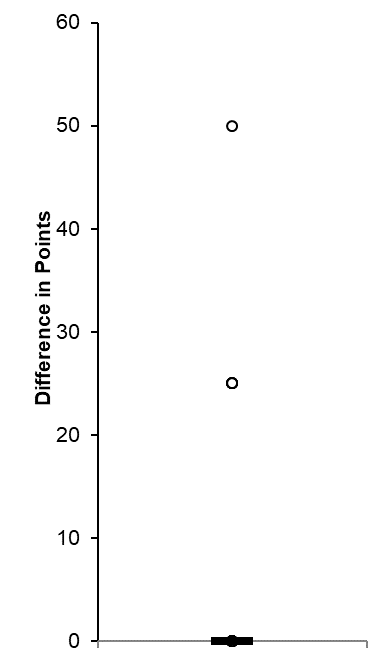


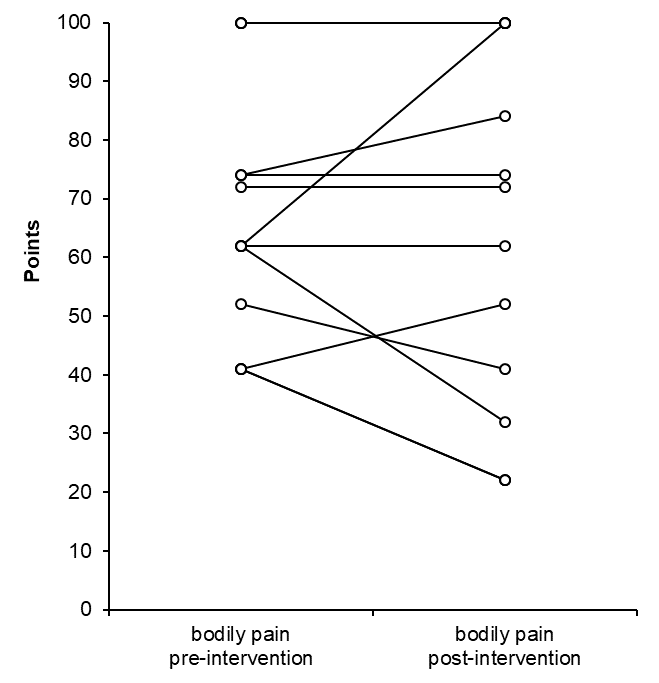

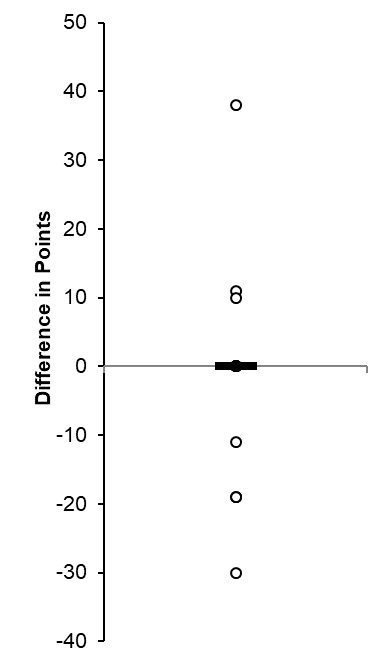

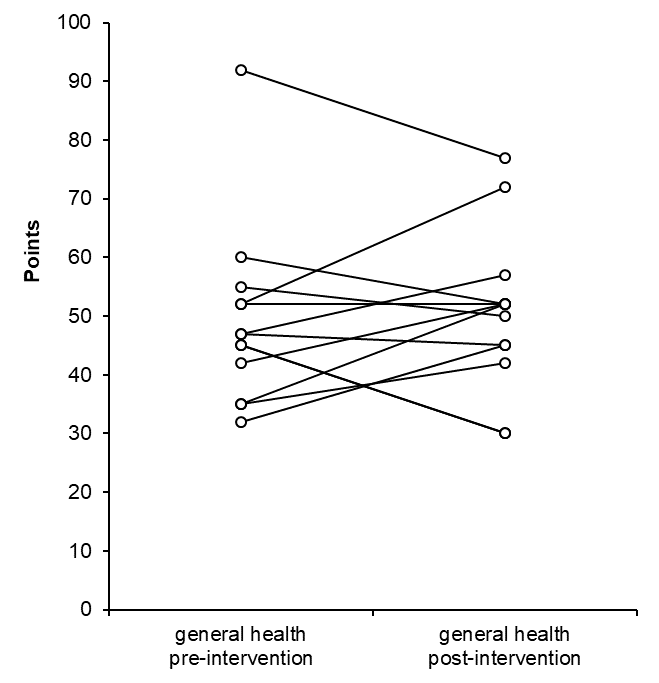

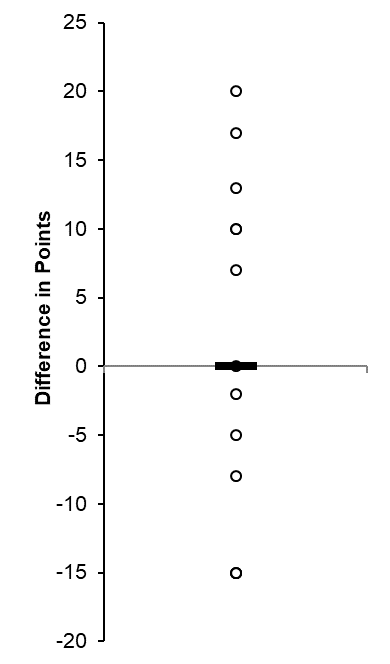


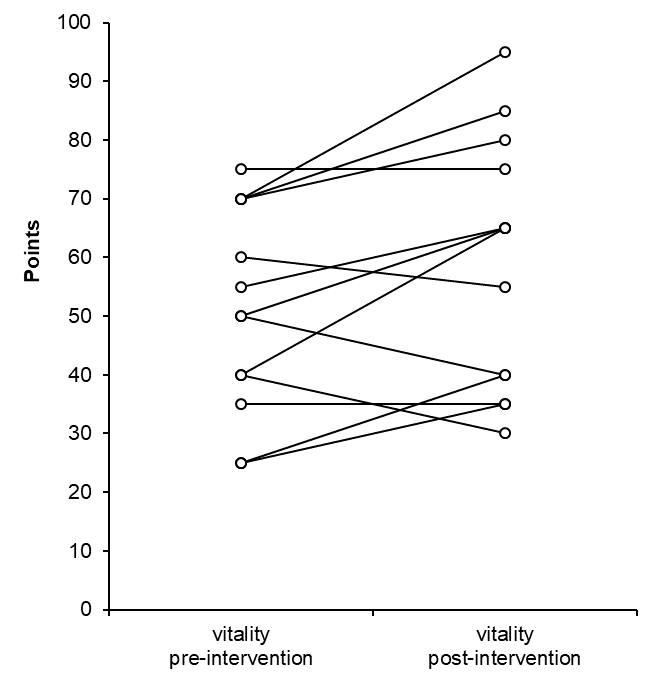

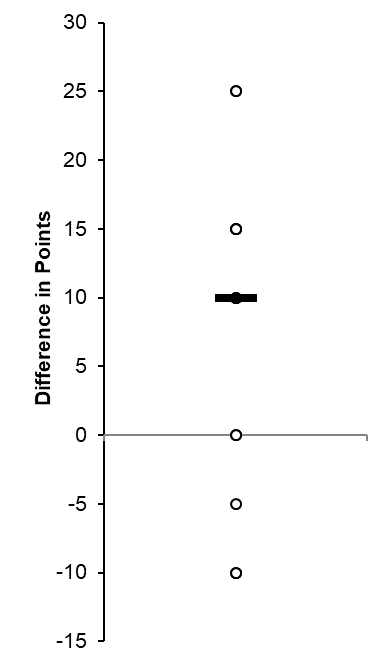

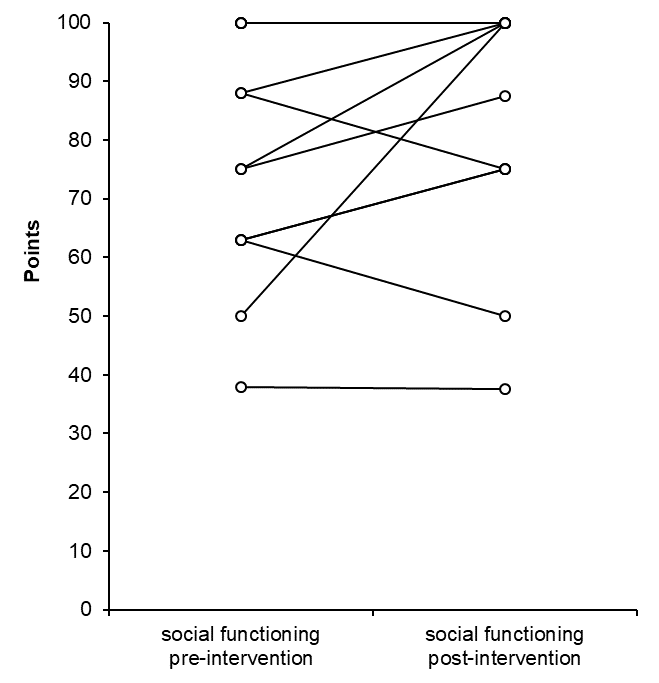

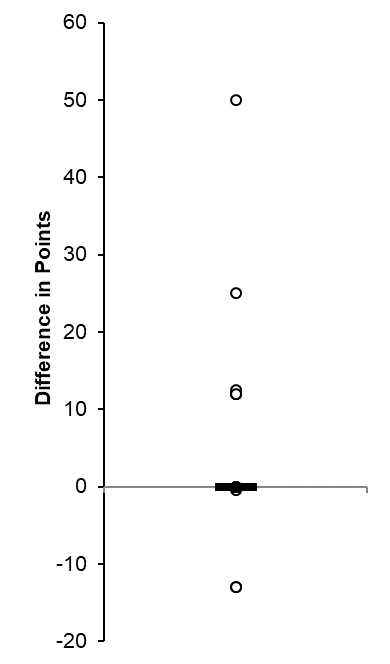


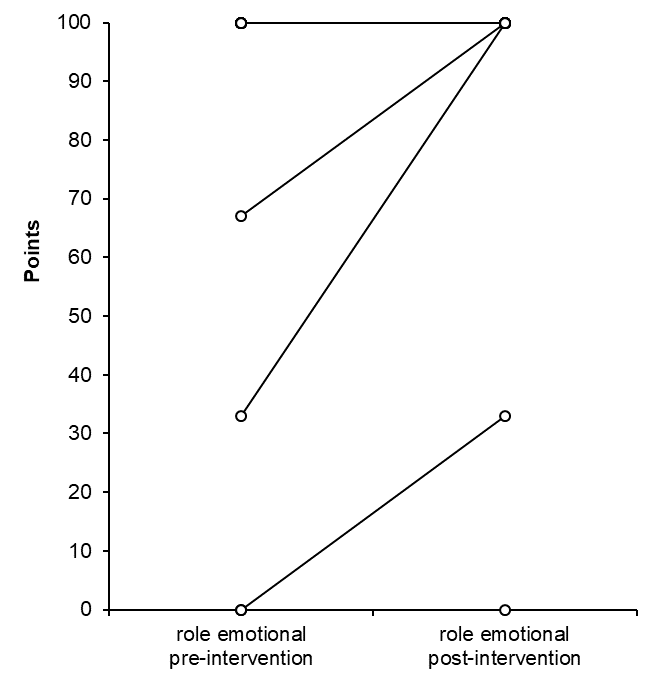

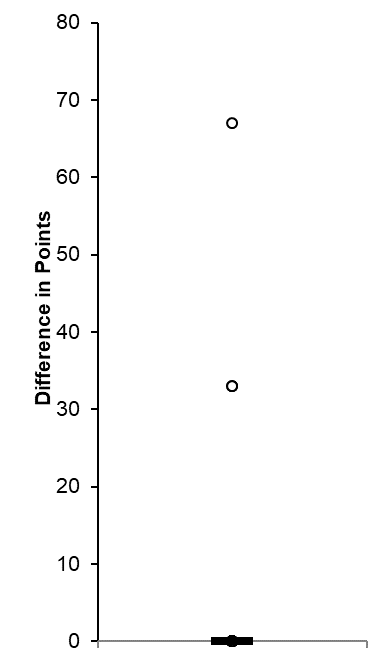

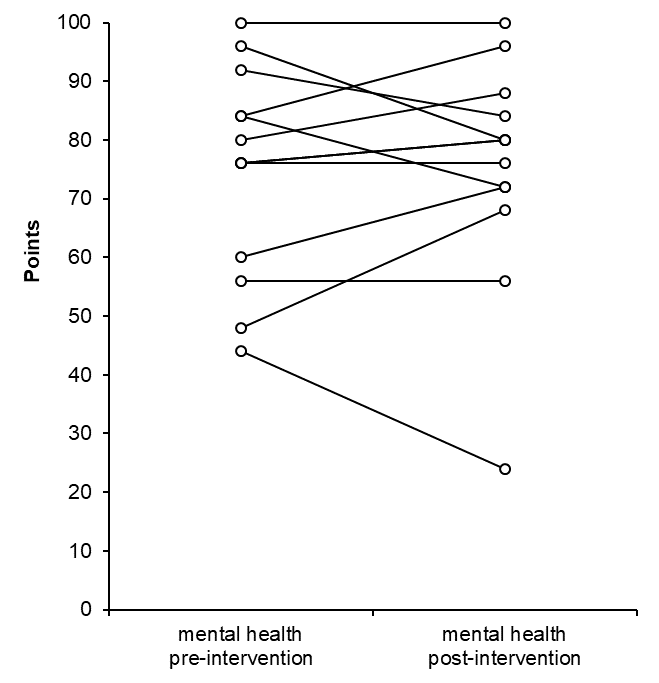

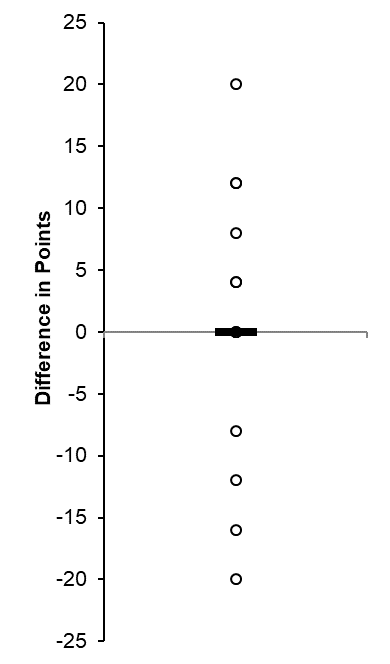


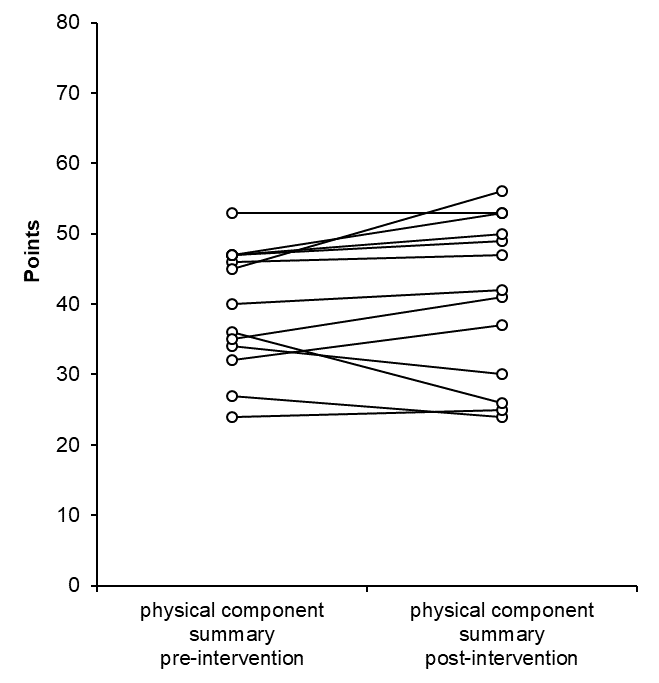

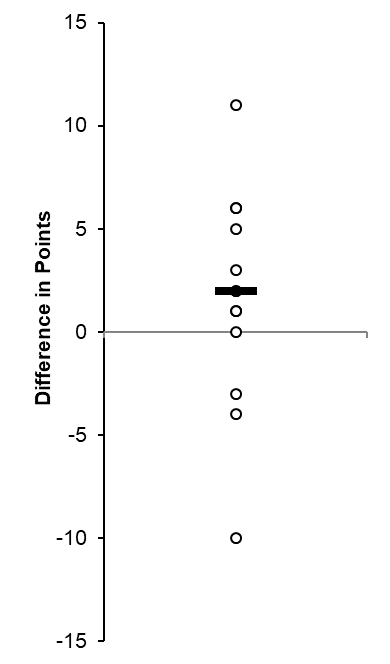

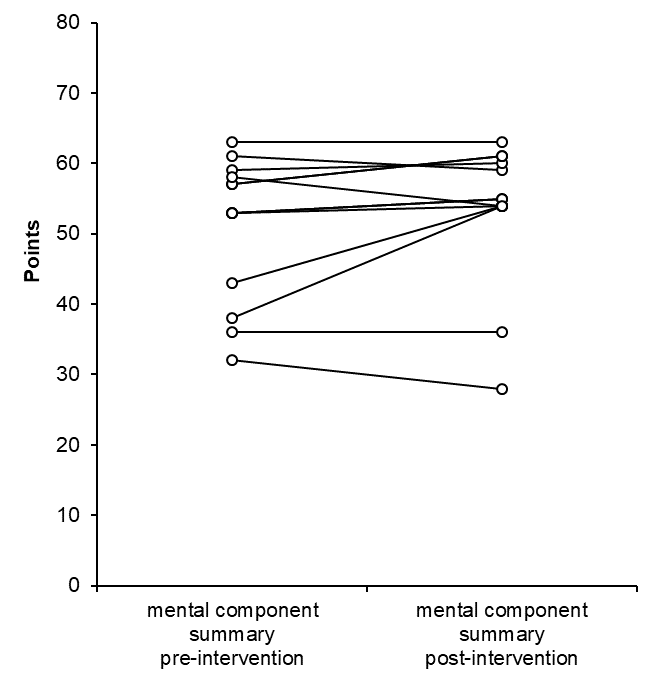

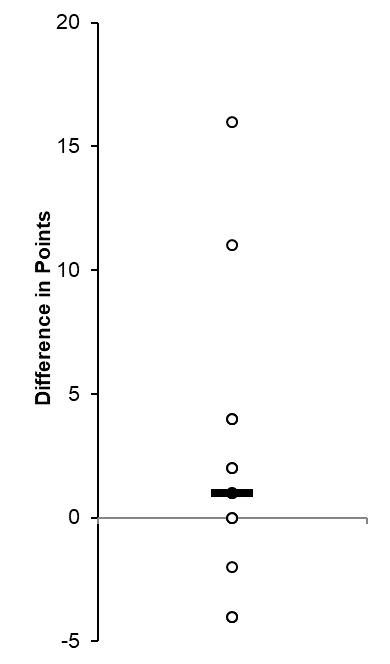


Legend: the solid lines show the median difference.

**Results of the Myositis Activity Profile (MAP)**


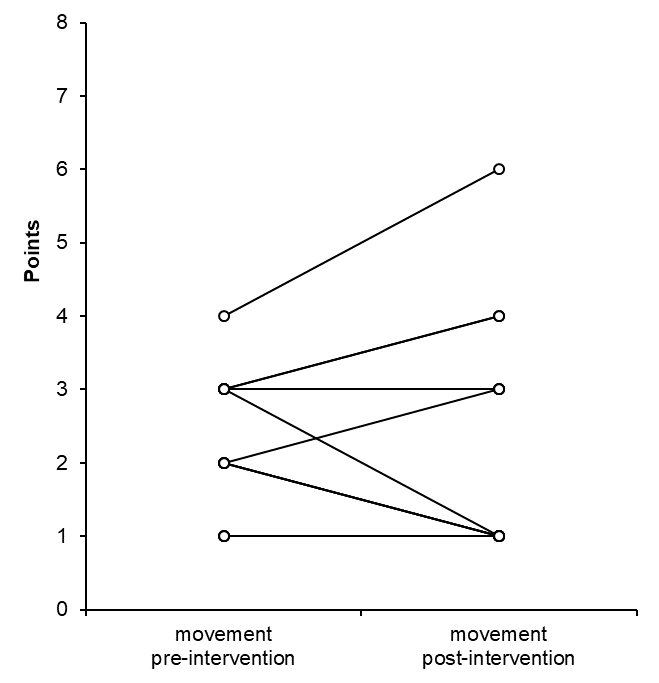

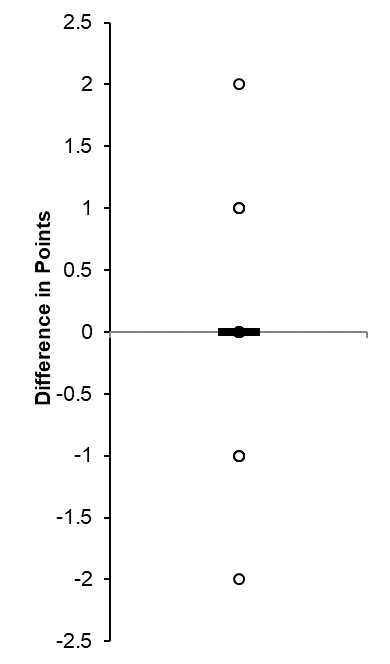

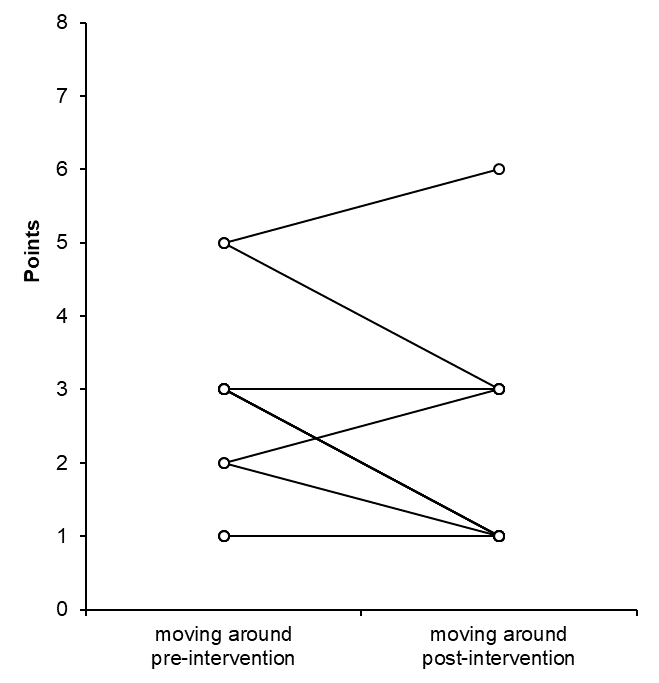

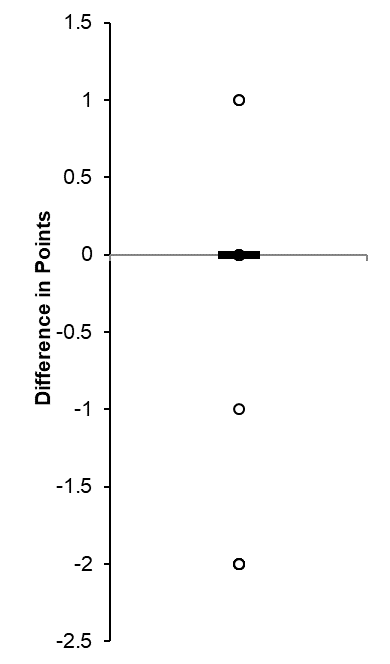


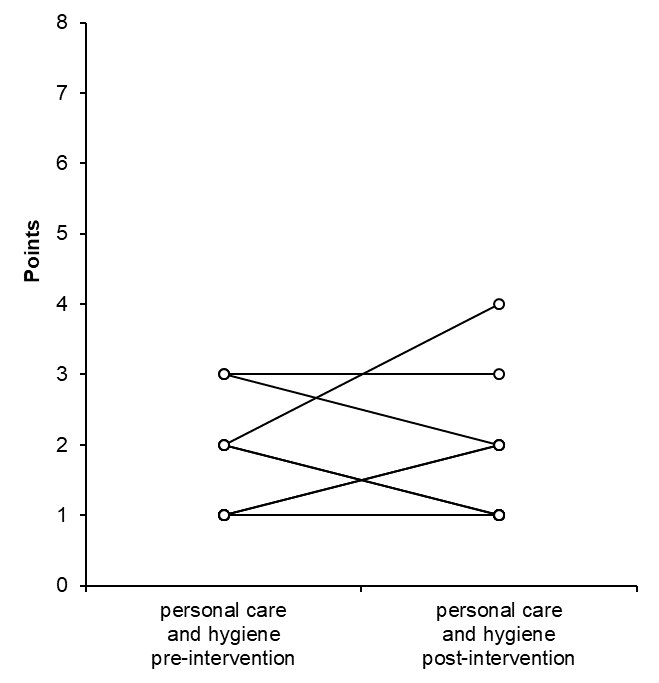

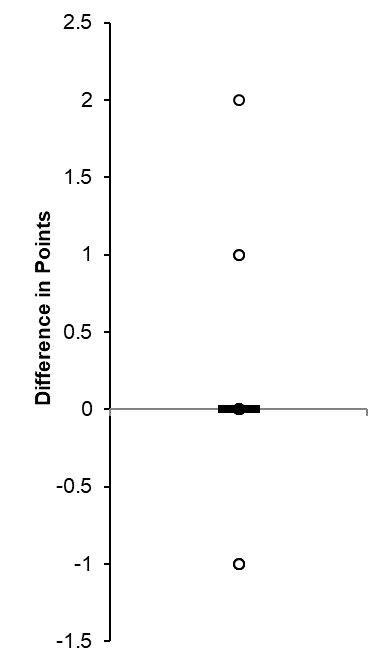

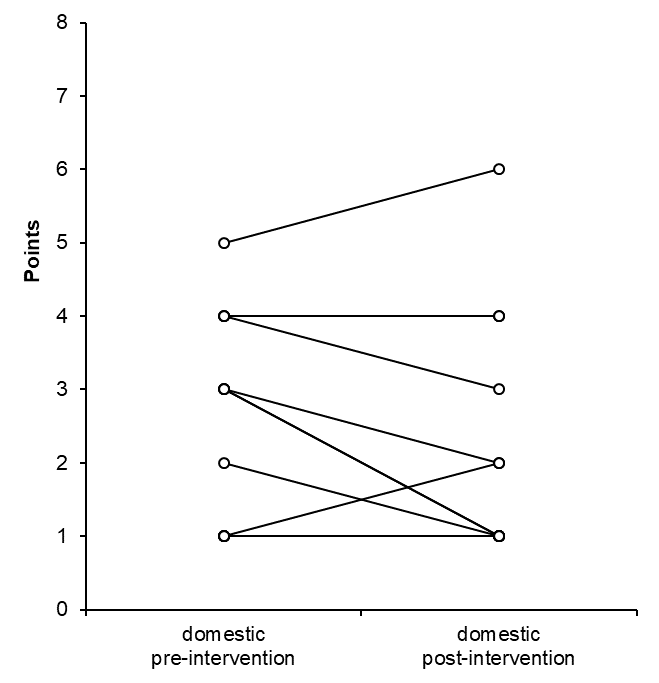

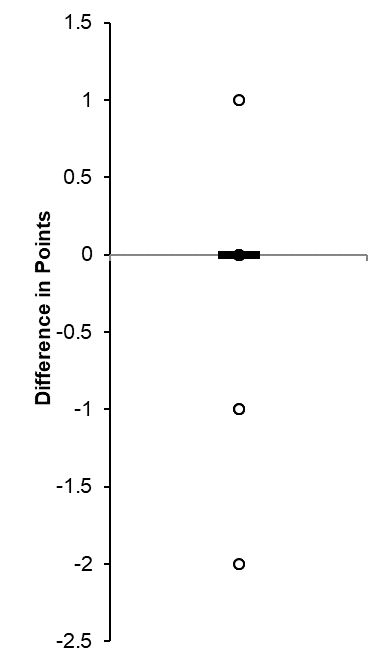


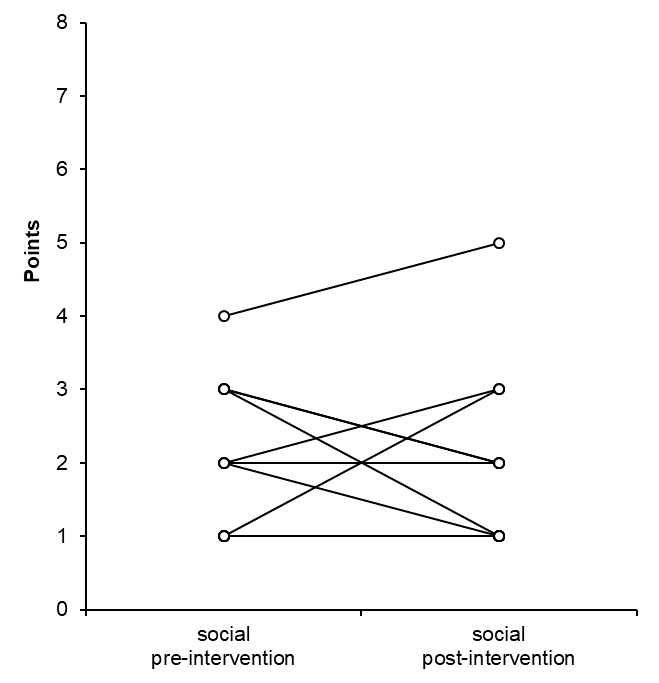

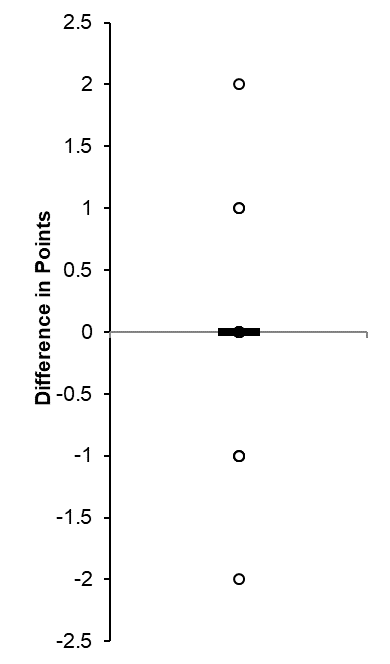

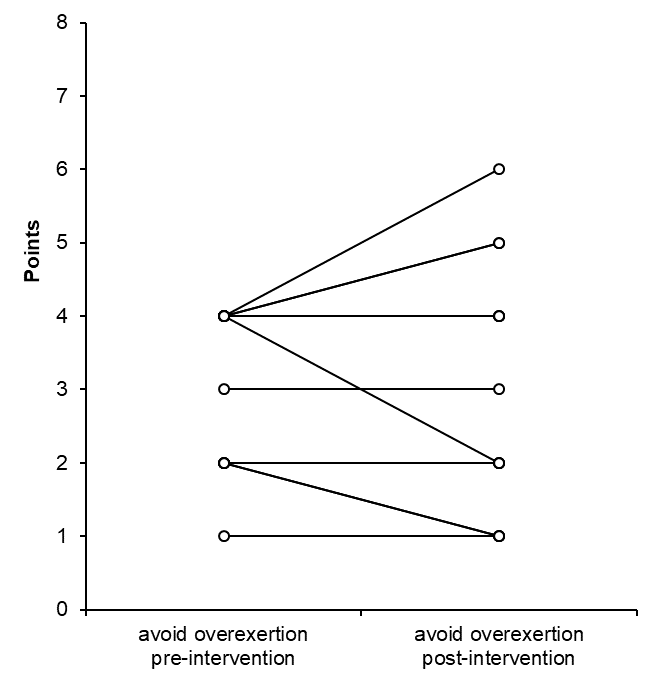

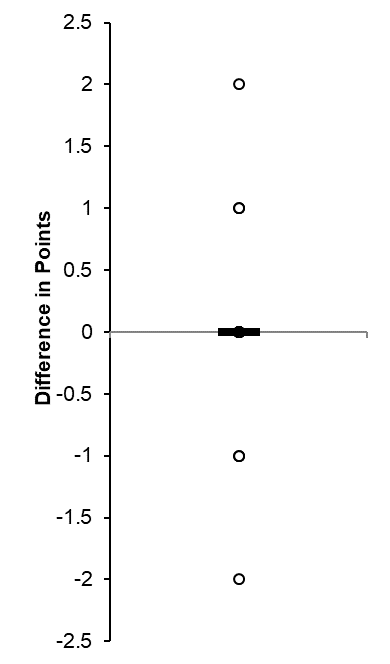


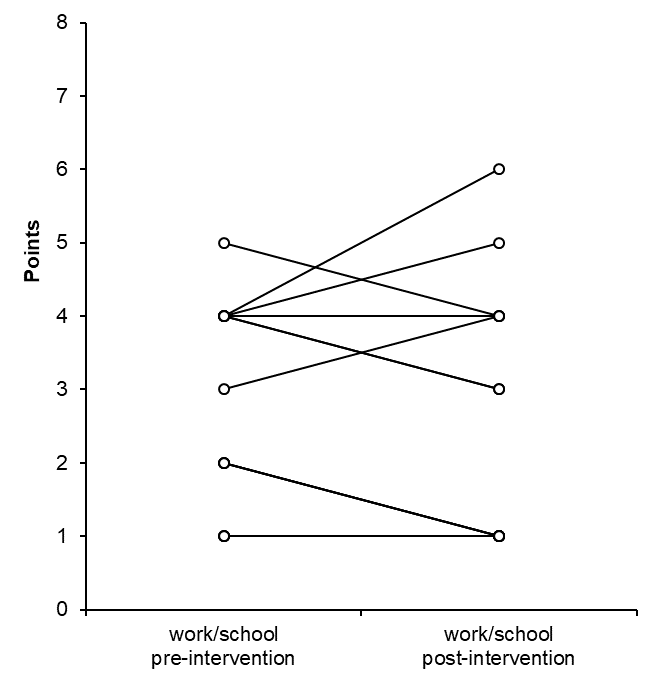

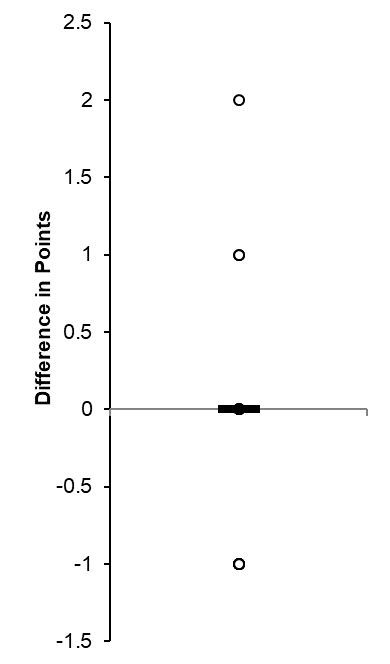

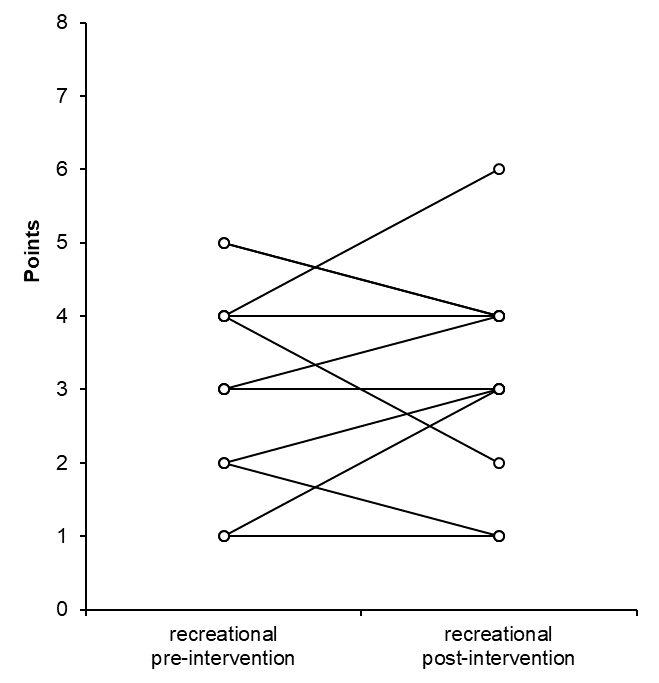

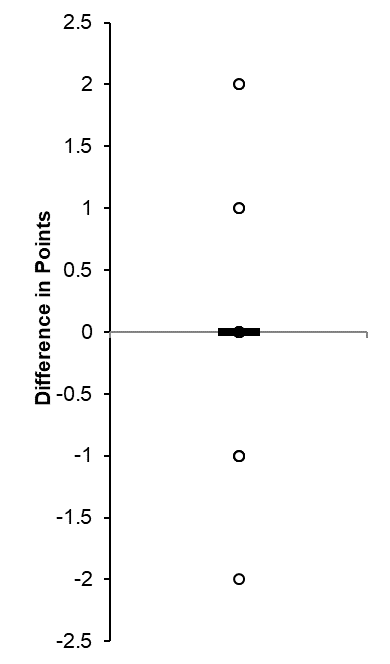


Legend: the solid lines show the median difference.

**Results of Stanford Health Assessment Questionnaire Disability Index (HAQ)**


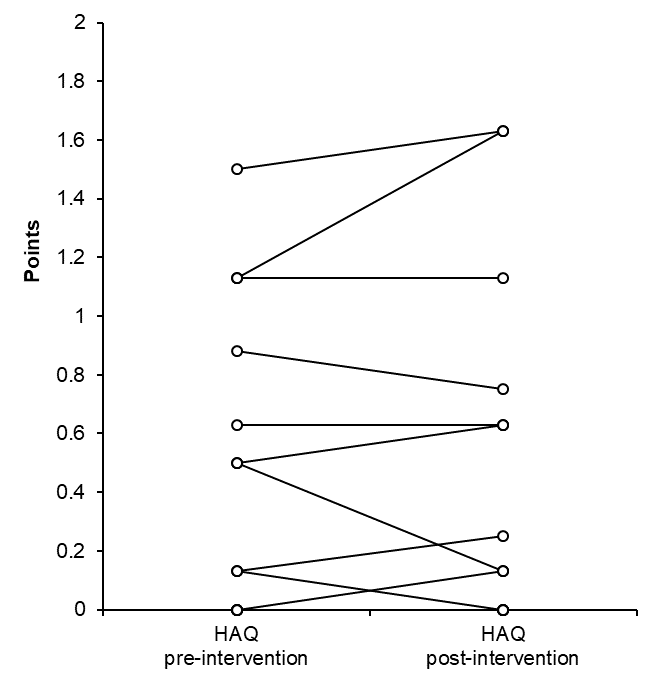

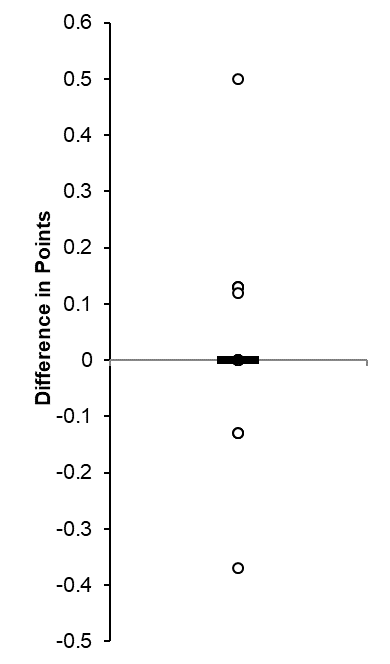


Legend: the solid lines show the median difference.
